# Chromosomal Instability in Near-Diploid Colorectal Cancer: A Link between Numbers and Structure

**DOI:** 10.1371/journal.pone.0001632

**Published:** 2008-02-20

**Authors:** Martine Muleris, Alexandra Chalastanis, Nicolas Meyer, Marick Lae, Bernard Dutrillaux, Xavier Sastre-Garau, Richard Hamelin, Jean-Francois Fléjou, Alex Duval

**Affiliations:** 1 INSERM, UMRS_762, CDR Saint-Antoine, F-75012, Paris, France; 2 UPMC Univ Paris 06, UMRS_762, CDR Saint-Antoine, F-75012, Paris, France; 3 Laboratoire de biostatistique et informatique médicale, Faculté de Médecine, Strasbourg, France; 4 Département de Santé Publique / CHU Strasbourg, Strasbourg, France; 5 Service de Pathologie, Département de Biologie des Tumeurs, Institut Curie, Paris, France; 6 UMR 5202, OSEB, Museum National d'Histoire Naturelle, Paris, France; 7 AP-HP Hôpital Saint-Antoine, Service d’Anatomie Pathologique, F-75012, Paris, France; University of Hong Kong, China

## Abstract

Chromosomal instability (CIN) plays a crucial role in tumor development and occurs mainly as the consequence of either missegregation of normal chromosomes (MSG) or structural rearrangement (SR). However, little is known about the respective chromosomal targets of MSG and SR and the way these processes combined within tumors to generate CIN. To address these questions, we karyotyped a consecutive series of 96 near-diploid colorectal cancers (CRCs) and distinguished chromosomal changes generated by either MSG or SR in tumor cells. Eighty-three tumors (86%) presented with chromosomal abnormalities that contained both MSGs and SRs to varying degrees whereas all 13 others (14%) showed normal karyotype. Using a maximum likelihood statistical method, chromosomes affected by MSG or SR and likely to represent changes that are selected for during tumor progression were found to be different and mostly mutually exclusive. MSGs and SRs were not randomly associated within tumors, delineating two major pathways of chromosome alterations that consisted of either chromosome gains by MSG or chromosomal losses by both MSG and SR. CRCs showing microsatellite instability (MSI) presented with either normal karyotype or chromosome gains whereas MSS (microsatellite stable) CRCs exhibited a combination of the two pathways. Taken together, these data provide new insights into the respective involvement of MSG and SR in near-diploid colorectal cancers, showing how these processes target distinct portions of the genome and result in specific patterns of chromosomal changes according to MSI status.

## Introduction

It has been demonstrated that chromosomes display non-random changes in cancer cells. These include structural rearrangements (SRs), e.g. deletions, amplifications or translocations that arise from breaks in DNA, as well as alterations in the number of intact chromosomes, known as whole-chromosome missegregations (MSGs), originating from errors in cell division (mitosis). As a result of the accumulation of such processes, chromosomal instability (CIN) is known to play a key role in tumor development. However, little is known about the exact contribution of MSG and SR in CIN and whether they act synergistically during tumor progression. Although chromosomal rearrangement is a well-documented process associated with tumorigenesis, the contribution of whole-chromosome aneuploidy to tumor development is still the subject of controversy. Colorectal cancers (CRCs) have been classified into two major molecular subtypes: CIN and MSI (for “microsatellite instability”, also called MIN). MSI CRCs account for approximately 15–20% of sporadic colorectal cancers. It is a well-defined subtype that results from a loss of DNA mismatch repair (MMR) function, secondary to inactivation of MMR genes. By failing to repair spontaneous errors that occur during replication, these tumors accumulate frameshift mutations that affect tumor suppressor genes containing coding repeat sequences [1]. MSI tumors are believed to be near-diploid with few, if any, karyotypic abnormalities. Conversely, CIN was found to occur in non-MSI cancers (or MSS for “microsatellite stable”) that represent the great majority of CRCs and are proficient for mismatch repair. Although observed in about 80% of sporadic colorectal tumors, the CIN phenotype is more poorly defined than MSI. Originally used to describe tumors that display a high degree of intercellular heterogeneity in chromosome number, ascertained by counts for a restricted set of chromosome-specific centromeres [2], CIN was further employed to describe cancers with either aneuploid or polyploid DNA content as measured by cytometry or cytogenetics, or multiple gains or deletions of chromosomes or chromosome arms, or frequent losses of heterozygosity (LOH). At present, there is no consensus for the experimental approach to be used or the minimum rate of chromosomal instability required to define CIN tumors. This results in much current confusion in the literature regarding the relationship between MSI and CIN following the method used to estimate CIN in such CRCs. Although MSI and CIN were considered mutually exclusive, both our previous data and recent studies suggest that some MSI tumors may also show evidence of CIN, although the extent and nature of this overlap remains to be determined [3]–[8].

Cytogenetics provides a morphological approach to CIN that in comparison to molecular studies allows to easily distinguish MSGs from SRs. Here, we used this approach to more precisely characterize CIN in a consecutive series of 96 near-diploid colorectal primary tumors that were prospectively collected over a 10 year period. Near-diploid tumors were chosen because of the ambiguity involved in determining gains and losses in polyploid tumors that have undergone endoreduplication. Indeed, interpretation of all numerical chromosome changes observed in a triploid tumor for instance, is totally different whether such tumor is considered as a diploid tumor that has gained many chromosomes or that has undergone endoreduplication with further subsequent chromosome losses to reach triploidy. In the present work, near-diploid CRCs were defined as those with a number of chromosomes between 35 and 57, according to the International System for Cytogenetic Nomenclature [9]. They constitute 50.2% of our initial series of karyotyped CRCs (data not shown), which is consistent with prior studies that have shown the diploid fraction of CRCs to be around 40% ([10], for review). We investigated in detail the nature and targets of CIN in these tumors. By distinguishing chromosomal changes generated by MSG or SR, we were able to compile a list of chromosomes or chromosomal regions targeted by MSG and SR and to study how these processes were associated within tumors in generating CIN. We also looked for differences in the nature of CIN between MSI and MSS subtypes of CRC. New insights concerning the role of CIN in CRC were obtained that allowed us to propose a new perspective on carcinogenesis in near-diploid CRCs which takes into account both their cytogenetic and molecular features.

## Results

### Missegregations and structural rearrangements of chromosomes target distinct portions of the tumor genome in near-diploid CRCs

**Figure 1:**
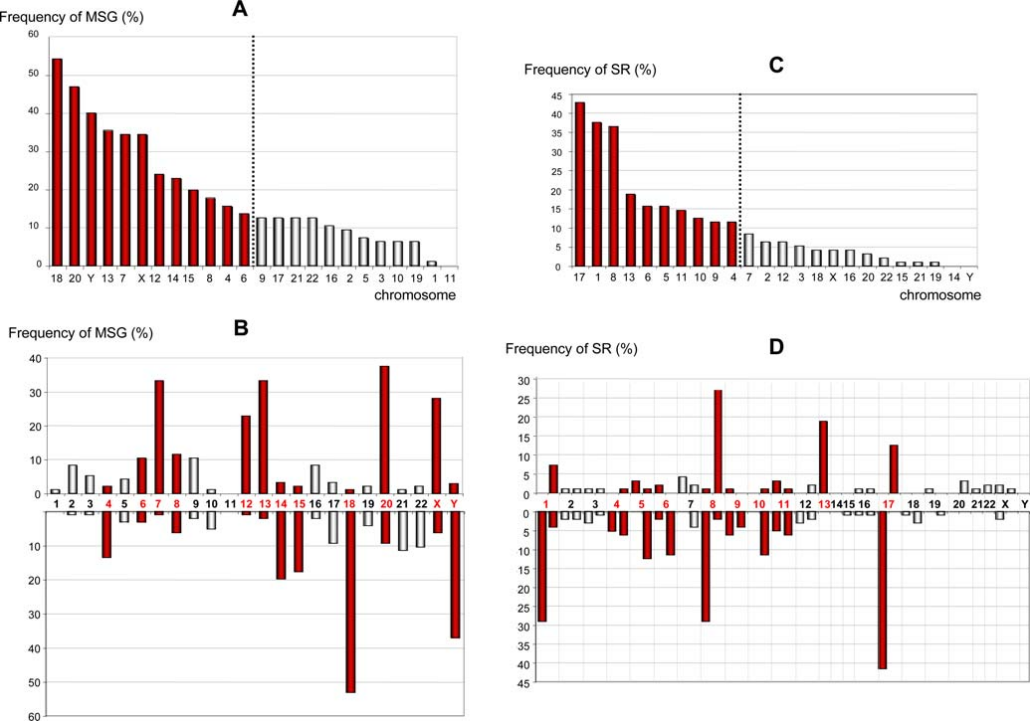
Involvement of chromosomes in missegregations (MSG) and structural rearrangements (SR) in our series of 96 near-diploid colorectal tumors. Frequencies of MSG (A) with their resulting gains and losses (B) and frequencies of SR (C) with their resulting gains and losses (D) are represented. The dotted line represents the cut-off value indicated by the likelihood statistical modeling that discriminates chromosomes that are likely to be selected for during tumor progression (red) from those constituting the background of chromosomal instability (grey). In B and D, each bar represents the percentage of loss (lower) or gain (upper) of a chromosome (B) or chromosome arm (D, p arm first, then q arm for non-acrocentric chromosomes).

**Figure 2:**
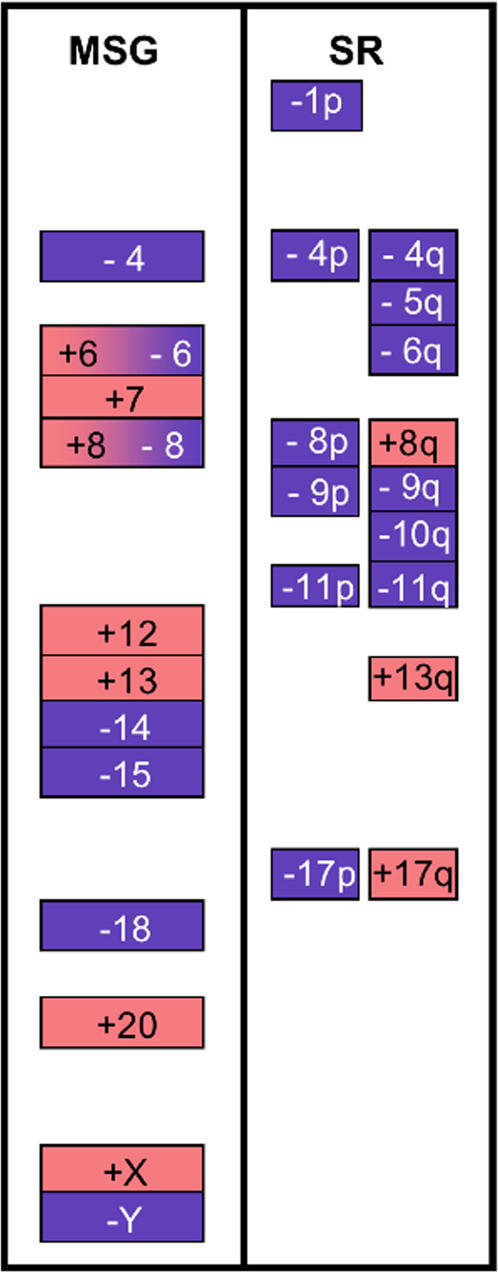
Most relevant chromosomal targets for missegregation (MSG) and structural rearrangements (SR) are mutually exclusive. Among the chromosomes or chromosome arms that are likely to be selected for during tumor progression according the likelihood modeling (see Figures 1B and 1D), only those for which a significative tendency towards either gain or loss were retained (Chi2 test, under the null hypothesis, the numbers of gains and losses should be distributed uniformly). Chromosomal losses (blue background) and gains (red background) are distinguished.

A series of 96 near-diploid colorectal tumors were analyzed among which 13 cases showed a normal karyotype. The total number of whole-chromosome gains was comparable to that of whole-chromosome losses (224 and 189 respectively, Chi2 = 2.96, p = 0.10). Whole-chromosome gains and losses involving individual chromosomes (Supplementary Table S2) were pooled and the distribution of missegregated chromosomes was tested using a likelihood statistical modeling. Frequencies of missegregation for individual chromosomes ranged from 0–54% (Figure 1A). The highest likelihood was observed for two groups containing 12 chromosomes each, with p1 = 0.42, p2 = 0.13 and alpha = 0.46. The first group comprised, by decreasing frequency of missegregation, chromosomes 18, 20, Y, 13, 7, X, 12, 14, 15, 8, 4 and 6. This group is likely to represent target chromosomes whose missegregation is selected for during tumor evolution. For most of these chromosomes, a clear tendency was observed towards either gain (chromosomes 7, 12, 13, 20 and X) or loss (chromosomes 4, 14, 15, 18 and Y) (Figure 1B). The second group is likely to represent the background of chromosomal instability occurring by MSG in colorectal tumors. The same approach was applied for chromosomes involved in structural rearrangements. Frequencies of rearrangements for individual chromosomes ranged from 0–42.7% (Figure 1C). The likelihood statistical modeling (p1 = 0.38, p2 = 0.07, alpha = 0.58) suggests that among the chromosomes involved in SRs, only chromosomes 17, 1, 8, 13, 6, 5, 11, 10, 9 and 4 are likely to represent target chromosomes that are selected for during tumor progression. For all of these chromosomes except 4, 9 and 11, a clear tendency was observed towards either chromosome arm gain (8q, 13q and 17q) or loss (1p, 5q, 6q, 8p, 10q and 17p) (Figure 1D). Among the total imbalances resulting from SRs, deletions were twice more frequent than gains (197 versus 101, respectively, Chi2 = 30.92, p<0.001, see Supplementary Table S3). A compilation of the chromosomal targets for MSG and SR is represented on Figure 2. Except for chromosomes 4, 6, 8 and 13, these were mutually exclusive (chromosomes 7, 12, 14, 15, 18, 20, X, Y for MSG compared to chromosomes 1, 5, 9, 10, 11, 17 for SR), highlighting the fact that these processes mainly target distinct portions of the tumor genome in near-diploid CRCs.

### Evidence for preferential associations among chromosome alterations

The total number of missegregated chromosomes per tumor ranged from 0–14 (mean = 4.53±3.08) and that of rearranged chromosomes from 0–15 (mean = 4.44±3.66). Using linear correlation analyses on the 83 tumors presenting an abnormal karyotype, the number of rearranged chromosomes was found not to be correlated with missegregation events within tumors (r = 0.20, p = 0.07). When whole-chromosome gains and losses were distinguished, the number of whole-chromosome gains and rearranged chromosomes was inversely correlated (r = −0.31, p = 0.004), whereas the increase in the number of whole-chromosome losses paralleled that of rearranged chromosomes (r = 0.69, p = 4.07 10^−13^).

**Figure 3:**
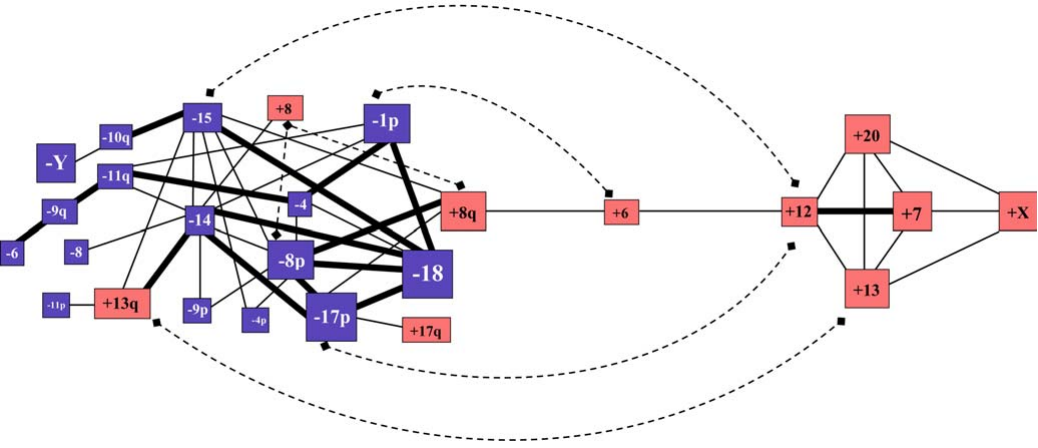
The two major chromosomal pathways in near-diploid CRCs. Schematic representation of the two-by-two association study presented in Supplementary Table S4. Positive (continuous lines) and negative (dotted lines) associations are represented by thick and thin lines indicating significant associations at p = 0.01 and 0.05 level, respectively. The size of each box is proportional to the frequency of the alteration, whole-chromosome or chromosome arm gains (red background), and chromosome losses or deletions (blue background) being distinguished. The preferential associations between alterations involving both arms of the same chromosome, such as -8p and +8q, or -17p and +17q, need to be interpreted with caution since they originate mostly from a single isochromosome formation event. For instance, gain of 17q seems to be a side effect of 17p deletion via isochromosome 17q formation (10/11 tumors with 17q gain also exhibited 17p loss whereas 17p loss alone was observed in an additional 30 tumors) whereas both 8p loss and 8q gain might provide tumor cells with a selective advantage since although associated in 20 tumors, these alterations were also observed independently in 8 and 7 tumors, respectively (see also Supplementary Figure S1). Some negative associations (between +13 and +13q, +8 and +8q, +8 and -8p) could also be due to the fact they involve the same chromosome.

We next investigated for possible preferential associations amongst the most frequent chromosome imbalances generated by either MSG or SR, i.e. those that were likely to be selected for in CRCs according to the likelihood method. A systematic analysis of two-by-two associations between 29 chromosomal imbalances (406 possibilities) demonstrated 51 associations (45 positive and 6 negative) that were significant in our tumor series (p = 0.05) (Supplementary Table S4). We are aware that considering that 406 analyses were performed, it could be expected that 20 out of these 51 significant associations were observed by chance alone (false positive) at the p = 0.05 level. Using a p = 0.01 level, 16 significant associations were found (Figure 3) that is four times more than the number of false positive expected, validating thus the existence of preferential associations. A p = 0.05 level was retained for the analysis. Amongst the 45 positive associations, most (84%) involved exclusively losses or gains (27 and 11, respectively) compared to 7 that showed gains mixed with losses (16%). Twenty (44.5%) showed associations of MSG with SR events compared to 15 (33.3%) and 10 (22.2%) that involved exclusively MSGs or SRs, respectively. By combining all results from two-by-two associations of chromosomal alterations observed in CRCs, two main groups of chromosomal abnormalities were delineated (Figure 3). The first group includes only whole-chromosome gains resulting from MSG (+13, +20, +7, +X, +12 and +6) whereas the second group mainly consists of chromosomal losses through MSG and SR (-18, -17p, -Y, -1p3, -8p, +8q, -14, +13q, -15, -4, +17q, +8, +6, -10q2, -11q, -9p, -8, -4p, -11p, -9q and -6, in decreasing order of occurrence). One minor group not related to the former two groups consisted of a preferential association between -4q and -5q.

### CIN according to MSI in colorectal cancers

The MSI status of 66 tumors, determined according to international criteria, showed 20 MSI and 46 MSS CRCs. Given that MSI tumors are known to be near-diploid CRCs, it resulted in an enrichment of MSI cases in our patients cohort that is restricted to near-diploid CRCs (30% i.e. 20/66 compared to the expected incidence of 15–20% in overall CRCs). This allowed us to obtain a consequent series of MSI tumors in order to compare their cytogenetic features to that of the most frequent MSS subtype. Clinicopathological characteristics of the tumors are presented in Table 1 and Supplementary Table S1. All variables except tumor location were equally distributed in the two subsets. Among the 10 tumors with normal karyotype whose MSI status was determined, 7 were MSI, confirming a high frequency of MSI in non-CIN CRCs. Global comparisons between MSS and MSI tumors showed that the latter group stayed very close to diploidy and exhibited significantly less MSGs (mean of 1.45 versus 5.82 for MSS tumors, p = 3×10^−10^) and SRs (mean 1.30 versus 5.02 for MSS tumors, p = 1.2×10^−6^) (Table 1). Of note, the few observed missegregations mainly consisted of whole-chromosome gains with very rare whole-chromosome losses whereas MSS tumors presented with more complex CIN including both MSG and SR leading to combined gains and losses of chromosomal material. Balanced rearrangements were relatively rare but did not significantly differ between MSI and MSS CRCs (Table 1).

**Table 1:** Comparison of MSI and MSS tumors: clinicopathological characteristics and frequency of chromosome alterations.

	MSS tumors	MSI tumors	p
Number of tumors	46	20	
Gender of the patient			
female	28	16	0.2*
male	18	4	
Mean age of the patient	65.65 (10.47)	62.75 (18.93)	0.5
Location of the tumor			
proximal	7	17	**3.4 10^−7^** *
distal	13	2	
rectum	26	1	
Astler Coller's stage of the tumor			
A	2	0	0.36 *
B	18	10	
C	13	8	
D	9	2	
undetermined	4	0	
Mean number of chromosomes (SD)	48.07 (4.25)	46.65 (0.88)	**0.03**
Mean number of missegregations (SD)	5.82 (3.06)	1.45 (1.67)	**3 10^−10^**
whole-chromosome losses	2.08 (2.24)	0.35 (0.81)	**2.04 10^−5^**
whole-chromosome gains	3.74 (3.40)	1.10 (1.59)	**6 10^−5^**
Mean number of rearranged chromosomes (SD)	5.02 (3.71)	1.30 (1.89)	**1.2 10^−6^**
Mean number of balanced rearrangements (SD)	0.41 (0.72)	0.3 (0.57)	0.50

All mean values are indicated with the standard deviation of the mean (SD). Comparisons were performed using Student *t* test or Chi2 (*).

### Classification of CRCs

Despite the existence of preferential two-by-two associations for some chromosomal alterations, we failed to observe a classification of tumors into different groups using unsupervised hierarchical cluster analysis based on chromosomal alterations with a significant confidence interval (bootstrap stimulation technique). This argues for the absence of available criteria delineating different subsets of near-diploid CRCs according to CIN.

## Discussion

Unlike some haematopoietic tumors or sarcomas, carcinomas are not characterized by translocation events that fuse an oncogene to an inappropriate promoter. Indeed in most epitheliomas, chromosomal instability proceeds through two major mechanisms, missegregation that results in aneuploidy through the gain or loss of whole-chromosomes, and unbalanced structural rearrangements (unbalanced translocations, deletions, isochromosomes, …) that lead to the loss and/or gain of chromosomal regions. We analyzed here a large series of near-diploid colorectal cancers by classical cytogenetics to distinguish chromosomal imbalances resulting from these two mechanisms. The use of the likelihood statistical modeling helped to define chromosome alterations that are likely to be selected for during tumor evolution and thus rise above the background of chromosomal instability. The outstanding features are that missegregation and structural rearrangements lead to chromosomal imbalances that are mostly mutually exclusive and combine to generate CIN in almost all tumors tested, suggesting that chromosomal disorders generated by MSG and SRs might have complementary rather than additive effects in CRCs.

The overall pattern of chromosomal imbalances we observed in near-diploid CRCs is consistent with previous reports that used other approaches such as CGH for the genome-wide assessment of DNA copy number in colorectal cancers (for review see [11]). However, the questions of how missegregations and rearrangements are distributed and how alterations are associated within tumors have rarely been addressed [12], [13]. Our data show that structural rearrangements accumulate concurrently with whole-chromosome losses during the progression of near-diploid CRCs. In contrast, whole-chromosome gains and rearrangements, are inversely correlated. Overall, preferential associations are found to delineate two pathways of chromosome alterations that favored the accumulation of either chromosomal losses or chromosome gains in colorectal tumor cells and that are not mutually exclusive. Of interest, MSG and SR highly cooperate in the pathway of chromosomal losses that is associated with the so-called LOH pathway since it leads to frequent chromosomal losses of the same loci as those identified by LOH in CRCs, e.g. 17p, 18 and others that contain tumor suppressor genes. In the other pathway, CIN leads exclusively to the accumulation of whole-chromosome gains through a missegregation process involving recurrently gains of chromosomes 20, 13, 7, X, 12 and 6. Negative associations for some imbalances are also observed (+12 and -15, +12 and -17p, and +6 and -1p) suggesting that combination of such abnormalities would be deleterious for tumor cells. All together, these data give a new insight on the way MSG and SR combine during the progression of near-diploid CRCs, suggesting that association of chromosomal imbalances rather than isolated chromosomal alterations would be of functional significance in this process.

Since microsatellite instability has been described as an alternative mechanism for colorectal cells to become malignant, we also compared cytogenetic alterations in near-diploid CRCs in relation to their microsatellite status. As expected, MSI tumors were preferentially located in the proximal colon. Interestingly, losses of whole-chromosomes were very rare in MSI CRCs and a net tendency for whole-chromosome gains was observed. Our data are consistent with some previous observations reporting that chromosome gains constitute a frequent feature of MSI CRCs. Indeed, early studies from our group using karyotyping demonstrated on a small sample of tumors that MSI CRCs displayed either a normal karyotype or chromosome gains with no or few rearrangements [3]. Subsequent studies on larger series of MSI tumors analyzed using chromosome comparative genomic hybridization (CGH) or array-based CGH (aCGH) have reported that gains of chromosomes and/or chromosome arms constitute the more frequent chromosome imbalances in MSI CRCs [4], [6]–[8]. However in a number of papers, MSI and CIN are considered to be mostly mutually exclusive. It is worth noting that in these papers, CIN has been estimated through LOH analysis. Indeed, since the early study of Thibodeau [14], several reports demonstrated that LOH events were rarely found in MSI CRCs ([15]–[18] for instance). Of interest, cytogenetics is a morphological approach suitable for the detection of both chromosomal gains and losses that are associated with CIN. Using this method, we report here mostly chromosomal gains and only few chromosomal losses in MSI CRCs, and conclude that such tumors are indeed CIN+ in most of cases (65%). It can therefore be assumed that the contradictory results that have been obtained following the use of LOH analysis are likely to be attributable, at least in part, to an inaccurate assessment of CIN in these studies. A larger series of MSI CRCs should now be analyzed in order to precise the most frequent chromosomal gains observed in these tumors. However, the coexistence of CIN and MSI should not be taken as the norm, especially since of 20 MSI tumors in our series, 7 (35%) exhibited a strictly normal karyotype.

Conversely to MSI CRCs that presented with a normal chromosome complement or mainly with chromosome gains, combinations of the two chromosomal pathways described above (chromosomal gains and losses) were found in MSS CRCs. Lastly, we found a subset of near-diploid MSS tumors that is microsatellite and chromosomal stable consistent with previous reports [4], [5], [16], [19]–[22]. Taking into account that 3 supplementary tumors with normal karyotype were of unknown microsatellite status, it could be expected that such a subset of tumors neither MSI nor CIN would constitute only a minor fraction of near-diploid CRCs i.e. 3 (3/96) to 6% (6/96) and thus maybe less than 3% of all CRCs. Although 13 out of 96 tumors in our series do not display chromosomal instability, it remains to be confirmed whether or not they are associated with LOH through cryptic mechanisms such as uniparental disomy or mitotic recombination.

CIN thus appears to play a role in both MSI and MSS colorectal tumors, but alternative patterns of chromosomal events might be selected for in near-diploid CRCs according to the presence or absence of MSI. Despite these observations, unsupervised hierarchical cluster analysis performed in our series was unable to separate tumors on the basis of chromosomal alterations. This discordance with the results reported by Trautmann et al. [7] may be due to a smaller number of tumors in their study (46 cases) and to the fact that these authors did not test the confidence of their cluster analysis results. It is consistent with the finding of no specific alterations for MSI tumors, the most recurrent gains in MSI tumors being also observed in MSS tumors.

**Figure 4:**
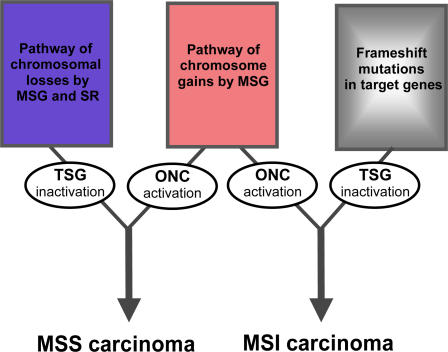
The role of chromosomal instability in near-diploid colorectal tumors according to MSI status. In MSS tumors, combination of the pathway of chromosome gains with that of chromosomal losses is necessary to target both oncogenes (ONC) and tumor suppressor genes (TSG), respectively. In MSI tumors, the pathway of chromosome gains is frequently observed whereas that of chromosomal losses is rarely found. In these tumors however, loss of function events are frequently observed as the consequence of frameshift mutations in coding microsatellite sequences. MSG, missegregation; SR, structural rearrangements.

The issue of the biological consequences of the non-random imbalances observed in colorectal carcinomas is still debated. It is generally considered that chromosomal losses confer an improved likelihood of inactivating tumor suppressor genes. However, current models of tumorigenesis generally fail to include a possible role for chromosome gains. More generally, chromosomal instability could provide a growth advantage to the cancer cell by causing extensive changes in gene expression via increased cell proliferation or decreased cell death. However, because of transcriptional regulation, the relationship between DNA copy number changes and perturbations in gene expression could be more complex than a simple dosage effect. Interestingly, Tsafrir et al. [23] recently demonstrated that expression of large groups of contiguous genes in MSS colorectal carcinomas varies in a coordinated way and reflects gain or loss of the corresponding chromosomal segment. Based on the differences we observed between MSS and MSI tumors, a putative model for carcinogenesis was proposed. It can be hypothesized that in MSS near-diploid tumors, combination of the pathway of chromosome gains with that of chromosomal losses is necessary to target both oncogenes surexpression and tumor suppressor genes inactivation respectively, whereas in MSI tumors, the observed gains of chromosomes likely result in an increased expression of putative oncogenes that would be complementary to the loss of function frameshift mutational events that affects tumor suppressor genes containing coding repeat sequences (Figure 4).

It is known that MSI CRCs stay near-diploid while a subset of MSS CRCs become polyploid, possibly through an endoreduplication process [24]. As already mentioned, polyploid CRCs could not be included in our analysis because of the ambiguity to distinguish chromosomal gains from losses in such CRCs. Nevertheless, it is noteworthy that our cytogenetic data on the subset of near-diploid CRCs are in agreement with the CGH profile from a meta-analysis comprising a total of 859 CRCs of all ploidy levels [11]. It has also to be noticed that our study cohort of patients is biased in terms of inclusion of familial cases. As stated in the supplementary table S1, 3 out of 96 patients with CRCs were identified as patients with familial adenomatous polyposis, so that it is unlikely that inclusion of these patients could have biased the overall results. Since there was neither family history data of the patients nor germline mutation screening for MLH1 or MSH2, it is likely that some individuals with an early onset MSI CRC enrolled in this study harbored hereditary non polyposis tumors. Even if cytogenetic data associated with MSI CRCs of either early (age ≤50 years at the time of surgery) or late onset (age >50 years) were here found to be quite similar with notably no significant difference concerning the number of tumors showing a normal karyotype (see supplementary tables S1, S2 and S3), it would be of interest to further investigate patients with familial CRCs identified on the basis of rigorous criteria.

The morphological approach we used to analyze the chromosomal instability in near-diploid colorectal carcinomas highlights the involvement of missegregative events affecting normal chromosomes, especially chromosome gains, in colorectal carcinogenesis. Our work is highly suggestive of an involvement of aneuploidy in CRCs through the selection of missegregation events that cooperate with other events generated in these tumors through chromosomal rearrangements or microsatellite instability. Further studies based on transcriptomic and proteomic approaches are now necessary to precise the functional consequences of the genomic pathways we have characterized here, taking into account the other molecular characteristics of CRCs.

## Materials and Methods

### Tumor specimens

Data were collected in the course of a systematic cytogenetic analysis of colorectal cancers operated at the Curie Institute, Paris. For the present study, we selected near-diploid tumors, that is with a mean number of chromosomes between 35 and 57 according to international criteria [9]. We used 96 tumors arising from 94 patients, with two patients having metachronous tumors. Three patients presented with typical syndrome of familial adenomatous polyposis. Because there was neither family history data of the patients nor germline mutation screening for MLH1 or MSH2, we cannot exclude the presence of hereditary non polyposis colorectal cancer in our series of tumors. Clinicopathological characteristics of patient age and sex, tumor location and Astler Coller staging are provided in Supplementary Table S1.

### Cytogenetic analysis

Cytogenetic analysis was performed on fresh tumors after surgery for most cases or on endoscopic biopsies in some instances according to our usual protocol [12]. Mechanical disaggregating of tissue was performed and short-term culture for 24–48 h was achieved in TC 199 medium supplemented with 20% of human serum and antibiotics. Cultures were harvested by a thymidine synchronization method in most instances. Thymidine was added to the culture medium at a final concentration of 0.3 mg/ml. After 17 hours, the culture medium was removed, cells were washed twice and incubated in fresh medium for 8 hours including a 3-hour treatment with colcemid. Cells were fixed and spread on glass slides and analysis was carried out on karyotyped cells after R-banding. In most tumors, cells with closely related abnormal chromosome complements were observed. Cells were considered as a clone when at least two had the same structural aberration or trisomy, or at least three had the same monosomy [9]. Comparison of clones led to the reconstruction of the chromosomal evolution of the tumor [25]. Only chromosome aberrations observed in clones and subclones were considered. Involvement of individual chromosomes in missegregation and rearrangements were scored separately, together with their resulting chromosomal imbalances (Supplementary Tables S2 and S3). Detailed information on structural abnormalities defined by band or sub-band is also provided (Supplementary Figure S1). Many rearrangements led to whole-arm imbalances. However, even for the rearrangements that resulted in loss or gain of only part of the arm, the chromosome arm was noted as imbalanced. For each tumor, the number of established karyotypes, the mean number of chromosomes, the number of balanced rearrangements and that of rearranged chromosomes and missegregations were recorded (Supplementary Tables S1, S2 and S3). In all the text we refer to missegregation as that of normal chromosomes.

### Determination of MSI status

MSI status was determined using pentaplex PCR or immunohistochemistry according to the material available. MSI determination was carried out using a modified version of the pentaplex PCR [26] in order to avoid the possibility of interference between different dyes during laser scanning. Primers were redesigned and shifted so that each amplification product would have a size differing by at least 20 bp from the others [27]. The five markers were co-amplified in a standard multiplex PCR with an annealing temperature of 55°C and were analyzed on an ABI PRISM 3100 Genetic Analyser according to manufacturer's instructions. Immunohistochemistry was performed as described [28] using mouse anti-human antibodies to MLH1 (dilution 1∶100, clone G168-728, Pharmingen, San Diego Calif., USA), MSH2 (dilution 1∶125, clone FE11, Calbiochem, Oncogene Research Products, Cambridge Mass., USA) and MSH6 (dilution 1∶100, clone 44, Becton Dickinson, Lexington, MA, USA).

### Statistical analysis

The distribution of chromosomes involved in missegregation and rearrangements was tested by likelihood statistical modeling according to [29], multiple gains being counted only once to avoid bias. All statistical tests were two-tailed. Chi2 was used to test the two-by-two associations between chromosome alterations using Yates' correction when necessary. In the comparison between MSI and MSS tumors, all mean values were compared using Student *t* test whereas the distribution of patient sex, tumor location and tumor staging was tested using Chi2. For clustering analysis, data were coded in binary form using “1” for AI and “−1” for normal informative locus. Different coding schemes were used but did not give substantially different results. Data were clustered using a two-way unsupervised clustering method, with uncentered correlation as similarity metrics for both genes and subjects value vectors. The average linkage was chosen as aggregation method. Computations were run under Gene Cluster 3.0. Cluster trees were produced using Java TreeView 1.0.4 (Eisen's Softwares®).

## Supporting Information

Table S1 Clinical, chromosomal and MSI data from 96 near-diploid colorectal tumors Case, tumor number; * patients with familial adenomatous polyposis; Age, patient's age (years); Gender, female (F) or male (M); A.C., Astler Coller staging; Loc, tumor location: left (L), proximal (P), rectum (R), sigmoid (S) or unknown (U); karyo, number of karyotypes established; Ch Nb, mean number of chromosomes; MSI, microsatellite instability status: instable (I), stable (S) or unknown (U)
(0.04 MB PDF)

Table S2 Karyo-array of the tumors: involvement of individual chromosomes in missegregations Chromosomes were coded as 2 if present in normal status (i.e. two copies for all autosomes, for X chromosome in females and one copy for X and Y chromosomes in males), as 1 or 0 (blue background) if one or two homologs were lost and as 3, 4 or 5 (red background) if one, two or three supernumerary copies were found. Case, tumor number; losses, number of whole-chromosome losses; gains, number of whole-chromosome gains; MSG, missegregation i.e. total number of whole-chromosomes losses and gains
(0.05 MB PDF)

Table S3 Karyo-array of the tumors: involvement of individual chromosomes in structural rearrangements and resulting chromosomal imbalances Chromosome arms were coded as 2 if present in normal status (i.e. two copies for all autosomes, for X chromosome in females and one copy for X and Y chromosomes in males), as 1 or 0 (blue background) if one or two copies copies were lost and as 3, 4 or 5 (red background) if one, two or three supernumerary copies were found. Case, tumor number; Rea, total number of rearranged chromosomes, a rearranged chromosome was counted only once even if present in more than one copy; Bal, number of balanced rearrangements; SR losses, number of chromosomal losses resulting from structural rearrangements; SR gains, number of chromosomal gains resulting from structural rearrangements, multiple gains of one chromosome arm were counted only once; p, short arm; q, long arm
(0.07 MB PDF)

Table S4 Analysis of the two-by-two associations between 29 chromosomal imbalances in the sample of 96 near-diploid colorectal tumors Observed (A) and theoretical (B) numbers of tumors that display (above the diagonal) or not (below the diagonal) each two-by-two association. Theoretical values were calculated under the hypothesis that associations occurred only by chance according to the relative frequency of each alteration. In C are indicated in each box the Chi2 values (cut-off value = 3.84, p<0.05) testing the observed number of tumors that display each association compared to the theoretical value. Forty-five associations were observed more frequently than expected by chance (positive associations), among them associations between chromosomal losses (blue background), chromosomal gains (pink background), or chromosomal gains and losses (purple background) are distinguished, and 6 were too rarely observed (negative associations, yellow background). NO: no object, *theoretical value is inferior to 1. The first row in italics in B indicates the frequency of each aberration.
(0.12 MB PDF)

Figure S1 R-banding schematic karyotype indicating the chromosomal imbalances resulting from the structural rearrangements identified in our sample of 96 near-diploid colorectal cancers. Red lines, gains; blue lines, losses; thick red lines, multiple gains; dotted blue lines, deletions within a chromosomal region that could not be accurately identified; numbers, case number.
(0.09 MB PDF)
