# Supplementary material for: Chromosomal Instability in Near-Diploid Colorectal Cancer: A Link between Numbers and Structure
Source: PLoS One. 2008 Feb 20;3(2):e1632. doi: 10.1371/journal.pone.0001632 (PMC2238794; doi:10.1371/journal.pone.0001632)
Supplement: Table S1 — Clinical, chromosomal and MSI data from 96 near-diploid colorectal tumors Case, tumor number; * patients with familial adenomatous polyposis; Age, patient's age (years); Gender, female (F) or male (M); A.C., Astler Coller staging; Loc, tumor location: left (L), proximal (P), rectum (R), sigmoid (S) or unknown (U); karyo, number of karyotypes established; Ch Nb, mean number of chromosomes; MSI, microsatellite instability status: instable (I), stable (S) or unknown (U) (0.04 MB PDF) [file pone.0001632.s001.pdf]

Case	Age	Gender	A. C.	Loc	Karyo	Ch Nb	MSI
1	58	F	C1	S	11	41	U
2	59	F	B2	R	21	51	S
3	56	M	B1	S	13	44	S
4	72	F	C1	R	12	47	S
5	72	F	C2	P	12	46	I
6	48	M	ND	R	3	45	U
7	48	F	B2	P	?	46	I
8	68	M	D	P	9	44	S
9	56	F	D	R	16	52	S
10	70	F	B2	P	8	46	I
11	66	F	B1	R	7	50	S
12	73	F	C2	P	11	49	I
13	64	M	C2	S	16	44	S
14	71	M	B2	S	5	44	S
15	71	M	B1	R	2	50	S
16	78	M	B2	S	10	49	U
17	62	F	B2	P	7	46	I
18	46	F	D	R	14	48	U
19	62	F	B2	P	9	46	I
20	70	M	D	R	11	41	U
21	55	F	B1	R	3	50	S
22	65	F	B1	P	8	47	I
23	29	M	C1	P	6	46	U
24	19	M	ND	L	6	46	U
25	82	F	B2	P	7	47	I
26	41	F	C2	R	19	49	U
27	75	F	B2	S	19	46	U
28	84	F	B2	S	10	44	S
29	66	F	C2	S	6	42	S
30	83	F	B2	R	8	44	S
31	75	F	B2	S	13	44	U
32	24	M	D	L	13	46	I
33	67	F	B2	S	12	57	S
34	29	F	C1	P	14	46	I
35	70	F	C2	R	12	48	S
36	52	F	C1	R	15	43	S
37	49	M	B2	R	18	49	S
38	58	M	D	R	2	47	U
39	83	F	B2	P	14	53	S
40*	31	F	C	R	14	40	U
41	56	M	B2	R	6	41	U
42	75	F	B1	R	40	42	S
43	78	M	ND	R	8	46	U
44	58	M	D	P	8	47	S
45	86	F	B1	P	11	46	I
46	75	F	B2	P	18	41	U
47	63	F	B2	P	10	47	U
48	72	M	B1	R	10	46	U
49	57	F	D	R	7	57	S
50	54	F	C1	R	6	46	S
51	71	F	B1	S	5	46	S
52	61	F	D	U	10	46	U
53	76	F	D	L	21	43	S
54	72	F	D	S	7	46	S
55	73	F	C1	P	7	46	S
56	57	F	D	L	7	42	U
57	57	M	A	R	15	51	S
58	29	F	B2	S	9	47	I
59	59	M	B2	L	11	46	S
60	48	F	A	P	1	49	S
61	69	M	ND	R	11	42	S
62	77	F	C1	P	12	47	I
63	72	F	C2	R	10	46	S
64	43	M	C2	P	29	47	I
65	48	F	B1	R	7	56	S
66	76	M	C2	R	11	44	U
67	78	M	ND	R	17	45	U
68	74	F	C1	R	24	45	U
69	67	M	D	P	13	46	I
70	84	M	C2	S	15	47	S
71	81	F	C1	P	12	47	I
72	84	F	A	R	14	47	U
73	64	F	D	S	8	47	S
74	52	F	C2	L	4	43	U

75	65	F	C1	R	11	50	S
76	43	F	D	L	8	47	U
77	59	M	B1	R	110	46	S
78	71	M	B1	R	7	51	S
79	79	F	C2	S	6	44	U
80	64	F	C	P	15	46	S
81	49	F	ND	S	15	53	S
82	80	M	D	R	7	46	S
83	76	F	B2	S	16	43	U
84	59	F	B1	P	2	46	I
85	54	F	D	P	19	49	S
86	80	M	ND	R	9	52	S
87	65	F	C1	R	16	48	I
88	72	M	B2	R	5	44	S
89	81	M	D	R	6	47	U
90	75	M	B2	P	9	48	I
91	82	F	ND	R	6	48	S
92*	57	M	C1	R	18	55	S
93	79	F	C1	S	11	43	U
94	86	F	C2	P	6	46	I
95	57	M	C2	R	8	58	S
96*	53	M	B2	R	19	46	U

Supplementary Table S1. Clinical, chromosomal and MSI data from 96 near-diploid colorectal tumors

Case, tumor number; * patients with familial adenomatous polyposis; Age, patient's age (years); Gender, female (F) or male (M); A.C., Astler Coller staging; Loc, tumor location: left (L), proximal (P), rectum (R), sigmoid (S) or unknown (U); karyo, number of karyotypes established; Ch Nb, mean number of chromosomes; MSI, microsatellite instability status: instable (I), stable (S) or unknown (U)
