# Supplementary material for: Chromosomal Instability in Near-Diploid Colorectal Cancer: A Link between Numbers and Structure
Source: PLoS One. 2008 Feb 20;3(2):e1632. doi: 10.1371/journal.pone.0001632 (PMC2238794; doi:10.1371/journal.pone.0001632)
Supplement: Table S2 — Karyo-array of the tumors: involvement of individual chromosomes in missegregations Chromosomes were coded as 2 if present in normal status (i.e. two copies for all autosomes, for X chromosome in females and one copy for X and Y chromosomes in males), as 1 or 0 (blue background) if one or two homologs were lost and as 3, 4 or 5 (red background) if one, two or three supernumerary copies were found. Case, tumor number; losses, number of whole-chromosome losses; gains, number of whole-chromosome gains; MSG, missegregation i.e. total number of whole-chromosomes losses and gains (0.05 MB PDF) [file pone.0001632.s002.pdf]

Case	losses	gains	MSG	1	2	3	4	5	6	7	8	9	10	11	12	13	14	15	16	17	18	19	20	21	22	X	Y
**TUMORS WITH MICROSATELLITE INSTABILITY**																											
5	3	0	3	2	2	2	2	2	1	2	2	2	2	2	2	2	2	2	2	2	1	2	2	2	2	1	*
7	0	0	0	2	2	2	2	2	2	2	2	2	2	2	2	2	2	2	2	2	2	2	2	2	2	2	*
10	0	0	0	2	2	2	2	2	2	2	2	2	2	2	2	2	2	2	2	2	2	2	2	2	2	2	*
12	0	3	3	2	2	2	2	2	3	2	3	3	2	2	2	2	2	2	2	2	2	2	2	2	2	2	*
17	0	0	0	2	2	2	2	2	2	2	2	2	2	2	2	2	2	2	2	2	2	2	2	2	2	2	*
19	2	2	4	2	2	2	2	2	2	2	2	2	2	2	2	2	2	2	2	2	3	1	2	1	3	2	*
22	0	1	1	2	2	2	2	2	2	2	2	2	2	2	3	2	2	2	2	2	2	2	2	2	2	2	*
25	1	0	1	2	2	2	2	2	2	2	3	2	2	2	2	2	2	2	2	2	2	2	2	2	2	2	*
32	0	0	0	2	2	2	2	2	2	2	2	2	2	2	2	2	2	2	2	2	2	2	2	2	2	2	2
34	0	0	0	2	2	2	2	2	2	2	2	2	2	2	2	2	2	2	2	2	2	2	2	2	2	2	*
45	0	0	0	2	2	2	2	2	2	2	2	2	2	2	2	2	2	2	2	2	2	2	2	2	2	2	*
58	0	4	4	2	3	2	2	2	2	3	2	2	2	2	3	3	2	2	2	2	2	2	2	2	2	2	*
62	0	0	0	2	2	2	2	2	2	2	2	2	2	2	2	2	2	2	2	2	2	2	2	2	2	2	*
64	0	3	3	2	2	2	2	2	2	3	2	2	2	2	2	2	3	2	2	2	2	2	3	2	2	2	2
69	1	0	1	2	2	2	2	2	2	2	2	2	2	2	2	2	2	2	2	2	2	2	2	2	2	2	1
71	0	2	2	2	2	2	2	2	2	2	3	2	2	2	2	3	2	2	2	2	2	2	2	2	2	2	*
84	0	0	0	2	2	2	2	2	2	2	2	2	2	2	2	2	2	2	2	2	2	2	2	2	2	2	*
87	0	5	5	2	2	2	2	2	2	3	2	2	2	2	2	3	3	3	2	2	2	2	3	2	2	2	*
90	0	2	2	2	2	2	2	2	2	2	2	2	2	2	3	2	2	2	2	2	2	2	3	2	2	2	2
94	0	0	0	2	2	2	2	2	2	2	2	2	2	2	2	2	2	2	2	2	2	2	2	2	2	2	*
**TUMORS WITHOUT MICROSATELLITE INSTABILITY**																											
2	0	5	5	2	2	2	2	2	2	3	2	2	2	2	3	3	2	2	2	3	2	2	3	2	2	2	*
3	4	5	9	2	2	3	1	2	3	3	2	2	2	2	2	0	2	2	2	2	1	2	3	2	2	3	1
4	2	3	5	2	2	2	2	2	2	2	2	2	2	2	3	2	2	2	2	2	1	2	3	1	2	3	*
8	4	2	6	2	2	2	1	2	2	2	2	2	2	2	2	3	2	2	2	1	1	2	3	2	1	2	2
9	0	6	8	2	3	2	2	2	2	3	3	2	2	2	3	5	2	2	2	2	2	2	3	2	2	2	*
11	0	5	5	2	3	2	2	2	2	3	2	2	2	2	3	2	2	2	2	2	2	2	3	2	2	3	*
13	5	1	6	2	2	2	2	2	2	2	2	2	2	2	2	2	2	1	2	2	1	1	3	1	2	2	1
14	10	0	10	2	1	2	1	2	2	2	1	2	1	2	2	1	1	2	1	2	1	2	2	1	2	2	1
15	0	4	4	2	2	2	2	2	2	3	2	2	2	2	3	2	2	2	2	2	2	2	3	2	2	3	2
21	0	5	5	2	2	2	3	2	2	3	2	2	2	2	3	3	2	2	2	2	2	2	3	2	2	2	*
28	7	0	7	2	2	2	2	2	2	1	2	2	1	2	2	2	1	1	2	1	1	2	2	2	1	2	*
29	3	1	4	2	2	2	2	2	2	2	2	2	2	2	2	2	2	2	2	2	1	2	3	2	1	1	*
30	6	0	6	2	2	2	2	2	2	2	2	2	1	2	2	2	1	1	2	2	1	2	1	2	1	2	*
33	0	11	11	2	3	2	2	2	3	3	2	3	2	2	3	4	2	2	3	2	2	2	4	2	2	3	*
35	2	4	6	2	2	2	2	2	2	3	2	2	2	2	2	3	2	2	2	1	2	2	4	2	2	1	*
36	6	0	6	2	2	2	1	2	2	2	2	1	2	2	2	2	2	2	1	2	1	2	1	2	2	1	*
37	2	4	6	2	2	2	2	2	3	3	2	2	2	2	3	2	2	2	2	2	1	2	2	1	2	3	2
39	2	6	8	3	3	2	2	2	2	3	1	3	2	2	2	2	2	2	3	2	1	2	3	2	2	2	*
42	4	1	5	2	2	2	1	2	2	2	2	2	2	2	2	3	1	1	2	2	1	2	2	2	2	2	*
44	4	1	5	2	2	2	2	2	2	2	2	2	2	2	2	2	2	1	2	2	1	2	3	2	1	2	1
49	2	12	14	2	2	2	2	3	2	3	1	3	2	2	3	4	2	2	3	2	1	3	4	2	2	4	*
50	1	2	3	2	2	2	2	2	2	2	2	2	2	2	2	2	2	2	2	2	1	2	3	2	2	3	*
51	2	3	5	2	2	2	2	2	2	2	2	2	2	2	2	4	1	2	2	2	1	2	3	2	2	2	*
53	3	0	3	2	2	2	2	2	2	2	2	2	2	2	2	2	2	1	2	2	1	2	2	2	1	2	*
54	3	0	3	2	2	2	2	2	2	2	2	2	2	2	2	2	2	1	2	1	1	2	2	2	2	2	*
55	0	0	0	2	2	2	2	2	2	2	2	2	2	2	2	2	2	2	2	2	2	2	2	2	2	2	*
57	1	9	10	2	2	3	2	3	3	2	3	3	2	2	3	3	2	2	2	2	1	2	3	2	2	3	2
59	0	0	0	2	2	2	2	2	2	2	2	2	2	2	2	2	2	2	2	2	2	2	2	2	2	2	2
60	1	3	4	2	2	2	2	2	2	3	3	2	2	2	3	2	2	2	2	1	2	2	2	2	2	2	*
61	4	0	4	2	2	2	2	2	2	2	2	2	2	2	2	2	1	1	2	2	1	2	2	2	2	2	1
63	0	2	2	2	2	2	2	2	2	2	3	2	2	2	3	2	2	2	2	2	2	2	2	2	2	2	*
65	0	10	10	2	2	3	2	2	3	2	2	3	3	2	3	4	2	2	4	2	2	2	2	2	2	3	*
70	3	5	8	2	2	2	2	2	2	2	2	2	2	2	2	5	2	1	3	2	1	2	3	1	2	2	2
73	3	3	6	2	2	2	2	2	2	3	2	1	2	2	2	2	2	3	2	2	1	2	3	2	2	1	*

75	0	5	5	2	2	2	2	3	2	3	2	2	2	2	2	3	2	2	2	2	2	2	3	2	2	3	*
77	1	3	4	2	2	2	2	2	2	2	3	2	2	2	2	2	2	2	2	2	1	2	3	2	2	3	2
78	2	5	7	2	2	2	2	2	2	3	2	2	2	2	3	3	2	2	2	2	1	2	3	2	2	3	1
80	0	0	0	2	2	2	2	2	2	2	2	2	2	2	2	2	2	2	2	2	2	2	2	2	2	2	*
81	0	8	8	2	3	2	2	2	3	3	2	3	2	2	3	3	2	2	2	2	2	2	4	2	2	2	*
82	0	1	1	2	2	2	2	2	2	2	2	2	2	2	2	2	2	2	2	2	2	2	2	2	2	2	3
85	0	7	7	2	2	2	2	3	2	3	2	2	2	2	2	4	2	2	3	2	2	2	3	2	2	3	*
86	0	7	7	2	3	2	2	2	2	3	3	2	2	2	2	4	2	2	3	2	2	2	2	2	2	3	2
88	3	1	4	2	2	2	1	2	2	2	2	2	2	2	2	2	1	2	2	2	1	2	3	2	2	2	2
91	3	4	7	2	2	2	2	2	2	3	2	2	2	2	2	3	2	2	2	2	1	2	3	1	1	3	*
92	2	6	8	2	2	1	3	2	3	3	2	2	2	2	3	3	2	2	2	3	2	2	2	2	2	2	1
95	1	12	13	2	2	3	2	2	2	3	4	2	2	2	3	3	3	2	2	3	2	2	4	3	2	3	1
**TUMORS WITH UNKNOWN MICROSATELLITE STATUS**																											
1	5	0	5	2	2	2	2	1	2	2	1	2	2	2	2	2	1	1	2	2	1	2	2	2	2	2	*
6	3	1	4	2	2	2	2	2	2	2	1	3	2	2	2	2	1	2	2	2	1	2	2	2	2	2	2
16	3	2	5	2	2	2	2	2	2	3	2	2	2	2	2	2	2	2	2	2	1	2	1	2	2	3	1
18	1	4	5	2	2	2	2	2	2	2	2	3	2	2	2	3	2	2	2	2	1	2	3	2	2	3	*
20	5	0	5	2	2	2	1	2	2	2	2	2	2	2	2	2	2	2	2	1	1	2	2	1	2	2	1
23	0	0	0	2	2	2	2	2	2	2	2	2	2	2	2	2	2	2	2	2	2	2	2	2	2	2	2
24	0	0	0	2	2	2	2	2	2	2	2	2	2	2	2	2	2	2	2	2	2	2	2	2	2	2	2
26	1	6	7	2	2	2	2	2	2	3	2	2	2	2	3	3	2	2	2	2	2	2	4	1	2	3	*
27	2	3	5	2	2	2	2	2	2	2	2	2	2	2	2	4	1	2	2	2	1	2	3	2	2	2	*
31	4	2	6	2	2	2	2	2	2	2	2	2	2	2	2	2	1	1	2	2	1	3	1	2	2	3	*
38	2	3	5	2	2	2	2	2	2	3	2	2	2	2	2	4	1	2	2	2	1	2	2	2	2	2	2
40	7	0	7	2	2	2	2	2	2	2	1	2	2	2	2	2	1	1	2	1	1	1	1	2	2	2	*
41	5	1	6	2	2	2	1	2	2	3	2	2	2	2	2	2	2	0	2	2	1	1	2	2	2	2	2
43	2	2	4	2	2	2	2	2	2	2	2	2	2	2	2	3	1	2	2	2	1	2	2	2	3	2	2
46	4	1	5	2	2	2	1	2	1	2	2	2	2	2	3	2	1	2	2	2	1	2	2	2	2	2	*
47	1	2	3	2	2	2	2	2	2	2	2	2	2	2	2	2	2	2	2	2	1	2	3	2	2	3	*
48	2	2	4	2	2	2	2	2	2	2	2	3	1	2	2	2	2	2	2	2	1	2	2	2	2	3	2
52	0	0	0	2	2	2	2	2	2	2	2	2	2	2	2	2	2	2	2	2	2	2	2	2	2	2	*
56	4	1	5	2	2	2	1	2	2	2	2	2	2	2	2	2	1	2	2	2	1	2	3	2	1	2	*
66	4	2	6	2	2	2	2	2	2	3	2	2	2	2	2	2	1	2	2	2	1	2	1	2	2	3	1
67	3	2	5	2	2	2	2	2	1	2	2	2	1	2	2	3	2	2	2	2	2	2	2	2	2	3	1
68	5	4	9	2	2	3	1	1	3	2	2	2	2	2	2	3	2	2	2	2	1	2	1	1	2	3	*
72	3	2	5	2	2	2	2	2	2	2	2	2	2	2	2	3	2	1	2	1	1	2	2	2	2	3	*
74	7	1	8	2	2	2	1	1	2	2	2	2	2	2	1	3	2	2	2	1	1	2	2	1	1	2	*
76	1	1	2	2	2	2	2	2	2	3	2	2	2	2	2	2	2	2	2	2	2	2	2	2	2	1	*
79	3	0	3	2	2	2	2	2	2	2	2	2	2	2	2	2	1	1	2	2	1	2	2	2	2	2	*
83	2	1	3	2	2	2	1	2	2	2	3	2	2	2	2	2	2	2	2	2	1	2	2	2	2	2	*
89	3	1	4	2	2	2	2	2	2	3	2	2	2	2	2	2	2	1	2	2	1	2	1	2	2	2	2
93	2	0	2	2	2	2	2	2	2	2	2	2	2	2	2	2	2	1	2	2	1	2	2	2	2	2	*
96	4	4	8	2	3	2	2	2	3	3	2	2	2	2	2	2	1	2	3	2	1	2	1	2	1	2	2

Supplementary Table S2. Karyo-array of the tumors: involvement of individual chromosomes in missegregations

Chromosomes were coded as 2 if present in normal status (i.e. two copies for all autosomes, for X chromosome in females and one copy for X and Y chromosomes in males), as 1 or 0 (blue background) if one or two homologs were lost and as 3, 4 or 5 (red background) if one, two or three supernumerary copies were found.
Case, tumor number; losses, number of whole-chromosome losses; gains, number of whole-chromosome gains; MSG, missegregation i.e. total number of whole-chromosomes losses and gains
