# Supplementary material for: Chromosomal Instability in Near-Diploid Colorectal Cancer: A Link between Numbers and Structure
Source: PLoS One. 2008 Feb 20;3(2):e1632. doi: 10.1371/journal.pone.0001632 (PMC2238794; doi:10.1371/journal.pone.0001632)
Supplement: Table S3 — Karyo-array of the tumors: involvement of individual chromosomes in structural rearrangements and resulting chromosomal imbalances Chromosome arms were coded as 2 if present in normal status (i.e. two copies for all autosomes, for X chromosome in females and one copy for X and Y chromosomes in males), as 1 or 0 (blue background) if one or two copies copies were lost and as 3, 4 or 5 (red background) if one, two or three supernumerary copies were found. Case, tumor number; Rea, total number of rearranged chromosomes, a rearranged chromosome was counted only once even if present in more than one copy; Bal, number of balanced rearrangements; SR losses, number of chromosomal losses resulting from structural rearrangements; SR gains, number of chromosomal gains resulting from structural rearrangements, multiple gains of one chromosome arm were counted only once; p, short arm; q, long arm (0.07 MB PDF) [file pone.0001632.s003.pdf]

Case	Rea	Bal	SR loss	SR gain	1p	1q	2p	2q	3p	3q	4p	4q	5p	5q	6p	6q	7p	7q	8p	8q	9p	9q	10p	10q	11p	11q	12p	12q	13q	14q	15q	16p	16q	17p	17q	18p	18q	19p	19q	20p	20q	21q	22q	Xp	Xq	Yp	Yq
**TUMORS WITH MICROSATELLITE INSTABILITY**																																															
5	7	2	1	0	2	2	2	2	2	2	2	2	2	2	2	2	2	2	2	2	2	2	2	2	2	2	2	1	2	2	2	2	2	2	2	2	2	2	2	2	2	2	2	2	2	*	*
7	0	0	0	0	2	2	2	2	2	2	2	2	2	2	2	2	2	2	2	2	2	2	2	2	2	2	2	2	2	2	2	2	2	2	2	2	2	2	2	2	2	2	2	2	2	*	*
10	0	0	0	0	2	2	2	2	2	2	2	2	2	2	2	2	2	2	2	2	2	2	2	2	2	2	2	2	2	2	2	2	2	2	2	2	2	2	2	2	2	2	2	2	2	*	*
12	1	0	0	1	2	4	2	2	2	2	2	2	2	2	2	2	2	2	2	2	2	2	2	2	2	2	2	2	2	2	2	2	2	2	2	2	2	2	2	2	2	2	2	2	2	*	*
17	0	0	0	0	2	2	2	2	2	2	2	2	2	2	2	2	2	2	2	2	2	2	2	2	2	2	2	2	2	2	2	2	2	2	2	2	2	2	2	2	2	2	2	2	2	*	*
19	0	0	0	1	2	2	2	2	2	2	2	2	2	2	2	2	2	2	2	2	2	2	2	2	2	2	2	2	2	2	2	2	2	2	2	2	2	2	2	2	2	2	2	2	2	*	*
22	2	1	0	0	2	2	2	2	2	2	2	2	2	2	2	2	2	2	2	2	2	2	2	2	2	2	2	2	2	2	2	2	2	2	2	2	2	2	2	2	2	2	2	2	2	*	*
25	4	1	1	1	2	2	3	2	2	2	2	2	2	2	2	2	2	2	2	2	2	2	2	2	2	2	2	2	2	2	2	2	1	2	2	2	2	2	2	2	2	2	2	2	2	*	*
32	0	0	0	0	2	2	2	2	2	2	2	2	2	2	2	2	2	2	2	2	2	2	2	2	2	2	2	2	2	2	2	2	2	2	2	2	2	2	2	2	2	2	2	2	2	2	2
34	0	0	0	0	2	2	2	2	2	2	2	2	2	2	2	2	2	2	2	2	2	2	2	2	2	2	2	2	2	2	2	2	2	2	2	2	2	2	2	2	2	2	2	2	2	*	*
45	0	0	0	0	2	2	2	2	2	2	2	2	2	2	2	2	2	2	2	2	2	2	2	2	2	2	2	2	2	2	2	2	2	2	2	2	2	2	2	2	2	2	2	2	2	*	*
58	1	0	0	0	2	2	2	2	2	2	2	2	2	2	2	2	2	2	2	2	2	2	2	2	2	2	2	2	2	2	2	2	2	2	2	2	2	2	2	2	2	2	2	2	2	*	*
62	4	1	0	2	2	2	2	2	2	2	2	2	2	2	2	2	2	2	2	3	2	2	2	2	2	2	2	3	2	2	2	2	2	2	2	2	2	2	2	2	2	2	2	2	2	*	*
64	3	0	1	3	2	2	2	2	2	2	2	2	2	2	2	2	2	2	3	4	2	2	2	2	2	2	2	2	2	2	2	2	2	1	3	2	2	2	2	2	2	2	2	2	2	2	2
69	0	0	0	0	2	2	2	2	2	2	2	2	2	2	2	2	2	2	2	2	2	2	2	2	2	2	2	2	2	2	2	2	2	2	2	2	2	2	2	2	2	2	2	2	2	2	2
71	2	0	2	0	2	2	2	2	2	2	2	2	2	2	2	1	2	2	2	2	2	2	2	2	2	2	2	1	2	2	2	2	2	2	2	2	2	2	2	2	2	2	2	2	2	*	*
84	0	0	0	0	2	2	2	2	2	2	2	2	2	2	2	2	2	2	2	2	2	2	2	2	2	2	2	2	2	2	2	2	2	2	2	2	2	2	2	2	2	2	2	2	2	*	*
87	0	0	0	0	2	2	2	2	2	2	2	2	2	2	2	2	2	2	2	2	2	2	2	2	2	2	2	2	2	2	2	2	2	2	2	2	2	2	2	2	2	2	2	2	2	*	*
90	1	1	0	0	2	2	2	2	2	2	2	2	2	2	2	2	2	2	2	2	2	2	2	2	2	2	2	2	2	2	2	2	2	2	2	2	2	2	2	2	2	2	2	2	2	2	2
94	1	0	0	1	2	2	2	2	2	2	2	2	2	2	2	2	2	2	2	2	2	2	2	2	2	2	2	2	3	2	2	2	2	2	2	2	2	2	2	2	2	2	2	2	2	*	*
**TUMORS WITHOUT MICROSATELLITE INSTABILITY**																																															
2	3	0	2	0	2	2	2	2	2	2	2	2	2	2	2	2	2	2	2	2	2	2	2	1	2	2	2	2	2	2	2	2	2	2	2	1	2	2	2	2	2	2	2	2	2	*	*
3	7	1	2	1	2	2	2	2	2	2	2	2	2	2	2	2	2	2	1	3	2	2	2	2	2	2	2	2	2	2	2	2	2	1	2	2	2	2	2	2	2	2	2	2	2	2	2
4	4	1	1	0	1	2	2	2	2	2	2	2	2	2	2	2	2	2	2	2	2	2	2	2	2	2	2	2	2	2	2	2	2	2	2	2	2	2	2	2	2	2	2	2	2	*	*
8	2	0	2	0	1	2	2	2	2	2	2	2	2	2	2	2	2	2	2	2	2	2	2	2	2	1	2	2	2	2	2	2	2	2	2	2	2	2	2	2	2	2	2	2	2	2	2
9	2	0	1	1	1	2	2	2	2	2	2	2	2	2	2	2	2	2	2	2	2	2	2	2	2	2	2	2	2	2	2	2	2	2	2	2	2	2	2	2	2	2	2	3	5	*	*
11	4	0	3	2	2	2	2	2	2	2	2	2	2	2	1	2	2	2	1	3	3	2	2	2	1	2	2	2	2	2	2	2	2	2	2	2	2	2	2	2	2	2	2	2	2	*	*
13	7	1	3	1	2	2	2	2	2	2	2	1	2	2	2	2	2	2	2	2	2	2	2	1	2	2	2	2	2	2	2	2	2	1	2	2	2	2	2	2	2	2	2	2	2	2	2
14	15	0	4	1	1	2	2	2	2	2	2	2	2	2	2	2	2	2	2	2	2	1	2	2	2	1	2	2	2	2	2	2	2	1	3	2	2	2	2	2	2	2	2	2	2	2	2
15	0	0	0	1	2	2	2	2	2	2	2	2	2	2	2	2	2	2	2	2	2	2	2	2	2	2	2	2	2	2	2	2	2	2	2	2	2	2	2	2	2	2	2	2	2	2	2
21	1	0	1	0	2	2	2	2	2	2	2	2	2	2	2	2	2	2	2	2	2	2	2	2	2	2	2	2	2	2	1	2	2	2	2	2	2	2	2	2	2	2	2	2	2	*	*
28	8	0	2	1	2	2	2	2	2	2	2	2	2	2	2	2	2	2	0	1	2	2	2	2	2	2	2	2	4	2	2	2	2	2	2	2	2	2	2	2	2	2	2	2	2	*	*
29	7	0	6	0	1	2	2	2	1	2	1	2	2	1	2	2	2	2	1	2	2	2	2	2	2	2	2	2	2	2	2	2	2	1	2	2	2	2	2	2	2	2	2	2	2	*	*
30	9	0	4	1	2	2	2	2	2	2	2	2	2	2	2	1	2	2	1	3	1	2	2	2	2	2	2	2	2	2	2	2	2	1	2	2	2	2	2	2	2	2	2	2	2	*	*
33	2	0	2	1	2	4	2	2	2	2	2	2	2	2	2	2	2	2	2	3	2	2	2	2	2	2	2	2	2	2	2	2	2	1	2	2	2	2	2	2	2	2	2	2	2	*	*
35	3	0	1	1	1	2	2	2	2	2	2	2	2	2	2	2	2	2	2	3	2	2	2	2	2	2	2	2	2	2	2	2	2	2	2	2	2	2	2	2	2	2	2	2	2	*	*
36	11	0	6	1	1	2	2	2	2	2	2	2	2	1	2	1	2	2	1	2	2	2	2	2	1	2	2	2	3	2	2	2	2	1	2	2	2	2	2	2	2	2	2	2	2	*	*
37	4	0	2	2	2	2	2	2	2	2	2	2	2	2	2	2	2	2	1	3	2	2	2	2	2	2	2	2	2	2	2	2	2	1	3	2	2	2	2	2	2	2	2	2	2	2	2
39	5	0	1	4	2	2	2	2	2	2	2	2	2	3	2	2	2	2	2	2	2	2	2	2	2	2	1	3	3	2	2	2	2	2	2	2	2	2	2	2	2	2	2	4	2	*	*
42	9	0	5	2	1	2	2	2	2	2	2	2	2	2	2	2	2	2	1	3	2	1	2	2	2	1	2	2	2	2	2	2	2	1	2	2	2	2	2	2	3	2	2	2	2	*	*
44	12	3	4	4	2	4	2	2	2	2	2	2	2	2	2	2	2	1	1	3	2	2	2	1	2	2	2	2	3	2	2	2	2	1	2	2	2	2	2	2	2	3	2	2	2	2	2
49	7	0	3	1	2	1	2	2	3	1	2	2	2	2	2	1	2	2	2	2	2	2	2	2	2	2	2	2	2	2	2	2	2	2	2	2	2	2	2	2	2	2	2	2	2	*	*
50	3	1	2	1	2	2	1	2	2	2	2	2	2	2	2	2	2	2	2	2	2	2	2	2	2	2	2	2	3	2	2	2	2	1	2	2	2	2	2	2	2	2	2	2	2	*	*
51	9	1	4	1	2	1	2	2	2	2	2	2	2	1	2	2	2	2	1	2	2	2	2	2	2	2	2	2	2	2	2	2	2	1	3	2	2	2	2	2	2	2	2	2	2	*	*
53	6	0	5	1	1	2	2	2	2	2	2	1	2	1	2	2	2	2	2	2	2	2	2	1	2	2	2	2	2	2	2	2	2	1	3	2	2	2	2	2	2	2	2	2	2	*	*
54	8	2	3	2	1	2	2	2	2	2	2	1	2	2	2	2	2	2	1	5	2	2	2	2	2	2	2	2	3	2	2	2	2	2	2	2	2	2	2	2	2	2	2	2	2	*	*
55	0	0	0	0	2	2	2	2	2	2	2	2	2	2	2	2	2	2	2	2	2	2	2	2	2	2	2	2	2	2	2	2	2	2	2	2	2	2	2	2	2	2	2	2	2	*	*
57	2	0	0	0	2	2	2	2	2	2	2	2	2	2	2	2	2	2	2	2	2	2	2	2	2	2	2	2	2	2	2	2	2	2	2	2	2	2	2	2	2	2	2	2	2	2	2
59	0	0	0	0	2	2	2	2	2	2	2	2	2	2	2	2	2	2	2	2	2	2	2	2	2	2	2	2	2	2	2	2	2	2	2	2	2	2	2	2	2	2	2	2	2	2	2
60	1	0	0	0	2	2	2	2	2	2	2	2	2	2	2	2	2	2	2	2	2	2	2	2	2	2	2	2	2	2	2	2	2	2	2	2	2	2	2	2	2	2	2	2	2	*	*
61	7	2	3	2	2	2	2	2	2	2	2	2	2	2	2	2	2	3	1	3	2	2	2	1	2	2	1	2	2	2	2	2	2	2	2	2	2	2	2	2	2	2	2	2	2	2	2
63	2	0	2	0	2	2	2	2	2	2	2	2	2	2	2	1	2	1	2	2	2	2	2	2	2	2	2	2	2	2	2	2	2	2	2	2	2	2	2	2	2	2	2	2	2	*	*
65	6	1	1	3	2	3	2	2	2	2	2	2	2	2	2	2	4	3	2	2	2	2	2	2	2	2	2	2	2	2	2	2	2	2	2	2	1	2	2	2	2	2	2	2	2	*	*
70	2	0	2	2	1	3	2	2	2	2	1	3	2	2	2	2	2	2	2	2	2	2	2	2	2	2	2	2	2	2	2	2	2	2	2	2	2	2	2	2	2	2	2	2	2	2	2
73	6	1	1	1	2	2	2	2	2	2	2	2	2	2	2	2	2	2	2	2	2	2	2	2	1	2	2	2	5	2	2	2	2	2	2	2	2	2	2	2	2	2	2	2	2	*	*
75	5	0	2	2	2	2	2	4	2	2	2	2	2	2	2	2	2	2	2	2	2	2	2	2	2	2	2	2	2	2	2	2	2	1	3	2	1	2	2	2	2	2	2	2	2	*	*
77	3	0	4	1	1	2	1	2	2	2	2	2	2	2	2	1	2	2	2	2	2	2	2	2	2	2	2	2	2	2	2	2	2	1	3	2	2	2	2	2	2	2	2	2	2	2	2
78	1	0	1	0	2	2	2	2	2	2	2	2	2	2	2	2	2	2	2	2	2	2	2	1	2	2	2	2	2	2	2	2	2	2	2	2	2	2	2	2	2	2	2	2	2	2	2
80	0	0	0	0	2	2	2	2	2	2	2	2	2	2	2	2	2	2	2	2	2	2	2	2	2	2	2	2	2	2	2	2	2	2	2	2	2	2	2	2	2	2	2	2	2	*	*
81	3	1	1	1	2	2	2	2	2	2	2	2	2	1	2	2	2	2	2	3	2	2	2	2	2	2	2	2	2	2	2	2	2	2	2	2	2	2	2	2	2	2	2	2	2	*	*
82	9	0	5	3	1	2	2	2	2	2	2	2	2	1	2	2	4	2	1	3	1	2	2	0	2	2	2	2	3	2	2	2	2	2	2	2	2	2	2	2	2	2	2	2	2	2	2
85	11	1	6	1	2	2	2	1	1	2	2	2	2	2	2	1	2	2	1	3	2	1	2	2	2	2	2	2	2	2	2	2	2	1	2	2	2	2	2	2	2	2	2	2	2	*	*
86	2	0	2	1	1	2	2	2	2	2	2	2	2	2	2	2	2	2	2	2	2	2	2	2	2	2	2	2	2	2	2	2	2	1	3	2	2	2	2	2	2	2	2	2	2	2	2
88	10	1	7	1	1	2	2	2	2	2	2	2	2	1	2	1	2	1	1	3	2	2	2	2	2	1	2	2	2	2	2	2	2	1	2	2	2	2	2	2	2	2	2	2	2	2	2

91	2	0	0	2	2	2	2	2	2	4	2	2	2	2	3	2	2	2	2	2	2	2	2	2	2	2	2	2	2	2	2	2	2	2	2	2	2	2	2	2	2	2	2	2	2	*	*
92	9	1	0	5	2	2	2	2	2	2	2	2	4	2	2	2	2	2	2	6	2	2	2	4	2	2	2	2	2	2	2	2	2	2	4	2	2	2	2	2	4	2	2	2	2	2	2
95	2	0	0	3	2	3	2	2	2	2	2	2	2	2	3	2	2	2	2	2	2	2	2	2	2	2	2	2	2	2	2	2	2	2	2	2	2	3	2	2	2	2	2	2	2	2	2
TUMORS WITH UNKNOWN MICROSATELLITE STATUS																																															
1	9	4	3	3	2	2	2	2	2	2	2	2	2	2	2	1	2	2	2	2	2	2	2	2	3	2	1	2	3	2	2	2	2	1	2	2	2	2	2	2	2	2	3	2	2	*	*
6	10	0	5	1	1	2	2	2	1	2	2	2	2	2	1	2	3	2	2	2	2	2	2	2	2	2	2	2	2	2	2	2	2	1	2	2	2	2	2	2	2	2	2	1	2	2	2
16	7	1	4	3	1	2	2	2	2	2	1	2	2	2	2	2	2	2	1	5	2	2	2	2	2	2	2	2	2	2	2	2	2	1	3	2	2	2	2	2	2	2	5	2	2	2	2
18	3	0	3	2	2	2	2	2	2	2	2	2	3	1	2	2	2	2	1	5	2	2	2	2	2	2	2	2	2	2	2	2	2	1	2	2	2	2	2	2	2	2	2	2	2	*	*
20	9	0	6	1	1	2	2	2	2	2	2	2	2	1	2	1	4	1	1	2	2	2	2	1	2	2	2	2	2	2	2	2	2	2	2	2	2	2	2	2	2	2	2	2	2	2	2
23	0	0	0	0	2	2	2	2	2	2	2	2	2	2	2	2	2	2	2	2	2	2	2	2	2	2	2	2	2	2	2	2	2	2	2	2	2	2	2	2	2	2	2	2	2	2	2
24	0	0	0	0	2	2	2	2	2	2	2	2	2	2	2	2	2	2	2	2	2	2	2	2	2	2	2	2	2	2	2	2	2	2	2	2	2	2	2	2	2	2	2	2	2	2	2
26	2	1	0	0	2	2	2	2	2	2	2	2	2	2	2	2	2	2	2	2	2	2	2	2	2	2	2	2	2	2	2	2	2	2	2	2	2	2	2	2	2	2	2	2	2	*	*
27	3	0	3	4	1	3	2	2	2	2	2	2	3	2	2	2	2	2	1	3	2	2	2	2	2	2	2	2	2	2	2	2	2	1	3	2	2	2	2	2	2	2	2	2	2	*	*
31	6	2	2	2	2	2	2	2	2	2	2	2	2	2	2	2	2	2	1	3	2	2	2	2	2	2	2	2	3	2	2	2	2	1	2	2	2	2	2	2	2	2	2	2	2	*	*
38	2	0	2	1	2	2	2	2	2	2	2	2	2	2	2	2	2	2	1	4	2	2	2	2	2	2	2	2	2	2	2	2	2	1	2	2	2	2	2	2	2	2	2	2	2	2	2
40	11	0	4	1	1	2	2	1	2	2	2	2	2	1	2	2	2	2	2	2	2	2	2	2	1	2	2	2	3	2	2	2	2	2	2	2	2	2	2	2	2	2	2	2	2	*	*
41	6	1	2	0	1	2	2	2	2	2	2	2	2	2	2	2	2	2	1	2	2	2	2	2	2	2	2	2	2	2	2	2	2	2	2	2	2	2	2	2	2	2	2	2	2	2	2
43	4	1	2	1	1	2	2	2	2	2	2	2	2	2	2	2	2	2	2	2	2	2	2	2	2	2	2	2	5	2	2	2	2	1	2	2	2	2	2	2	2	2	2	2	2	2	2
46	14	2	5	2	1	2	2	2	2	2	2	2	2	2	2	2	2	2	2	2	2	2	2	2	2	1	2	2	3	2	2	1	2	1	2	2	2	2	2	2	4	2	2	1	2	*	*
47	5	0	4	0	2	2	2	2	2	2	2	1	2	1	2	2	2	2	2	2	2	1	2	2	2	2	2	2	2	2	2	2	2	1	2	2	2	2	2	2	2	2	2	2	2	*	*
48	7	1	2	0	2	2	2	2	2	2	2	2	2	2	2	1	2	2	2	2	2	2	2	2	2	2	2	2	2	2	2	2	2	1	2	2	2	2	2	2	2	2	2	2	2	2	2
52	0	0	0	0	2	2	2	2	2	2	2	2	2	2	2	2	2	2	2	2	2	2	2	2	2	2	2	2	2	2	2	2	2	2	2	2	2	2	2	2	2	2	2	2	2	*	*
56	7	2	3	1	1	2	2	2	2	2	2	2	2	2	2	2	2	2	2	2	1	2	2	2	2	2	2	2	4	2	2	2	2	1	2	2	2	2	2	2	2	2	2	2	2	*	*
66	7	1	4	4	1	2	2	2	2	2	2	2	2	2	2	2	2	2	1	3	1	2	2	2	3	2	2	2	4	2	2	3	2	1	2	2	2	2	2	2	2	2	2	2	2	2	2
67	3	1	2	0	2	2	2	2	2	2	2	2	2	2	2	2	2	2	2	2	2	2	2	2	2	2	2	2	2	2	2	2	2	1	2	2	1	2	2	2	2	2	2	2	2	2	2
68	8	1	2	3	2	2	2	2	2	2	2	2	2	2	2	2	2	2	1	3	2	2	2	2	3	4	2	2	2	2	2	2	2	1	2	2	2	2	2	2	2	2	2	2	2	*	*
72	5	0	0	1	2	2	2	2	2	2	2	2	2	2	2	2	2	2	2	3	2	2	2	2	2	2	2	2	2	2	2	2	2	2	2	2	2	2	2	2	2	2	2	2	2	*	*
74	9	0	4	0	1	2	2	2	2	2	2	2	2	2	2	2	2	2	1	2	1	2	2	1	2	2	2	2	2	2	2	2	2	2	2	2	2	2	2	2	2	2	2	2	2	*	*
76	5	0	4	1	2	2	2	2	2	2	2	1	2	1	2	2	2	2	2	2	2	2	2	2	2	1	2	2	4	2	2	2	2	1	2	2	2	2	2	2	2	2	2	2	2	*	*
79	4	0	3	1	1	2	2	2	2	2	2	2	2	2	2	2	2	2	2	2	2	2	2	1	2	2	2	2	3	2	2	2	2	1	2	2	2	2	2	2	2	2	2	2	2	*	*
83	4	0	3	0	1	1	2	2	2	2	2	2	2	2	2	2	2	2	2	2	2	2	2	2	1	2	2	2	2	2	2	2	2	1	2	2	2	2	2	2	2	2	2	2	2	*	*
89	6	0	3	1	2	2	2	2	2	2	1	2	2	2	2	2	2	2	1	3	2	2	2	2	2	2	2	2	2	2	2	2	2	1	2	2	2	2	2	2	2	2	2	2	2	2	2
93	4	0	4	1	2	2	2	2	2	2	1	2	2	2	2	2	2	2	1	3	2	2	2	1	2	2	2	2	2	2	2	2	2	1	2	2	2	2	2	2	2	2	2	2	2	*	*
96	9	0	5	1	2	1	2	2	2	2	2	1	2	2	2	2	2	2	1	2	1	2	2	2	2	2	2	2	2	2	2	2	4	1	2	2	2	2	2	2	2	2	2	2	2	2	2

Supplementary Table S3. Karyo-array of the tumors: involvement of individual chromosomes in structural rearrangements and resulting chromosomal imbalances

Chromosome arms were coded as 2 if present in normal status (i.e. two copies for all autosomes, for X chromosome in females and one copy for X and Y chromosomes in males), as 1 or 0 (blue background) if one or two copies copies were lost and as 3, 4 or 5 (red background) if one, two or three supernumerary copies were found.
Case, tumor number; Rea, total number of rearranged chromosomes, a rearranged chromosome was counted only once even if present in more than one copy; Bal, number of balanced rearrangements; SR losses, number of chromosomal losses resulting from structural rearrangements; SR gains, number of chromosomal gains resulting from structural rearrangements, multiple gains of one chromosome arm were counted only once; p, short arm; q, long arm
