# Supplementary material for: Chromosomal Instability in Near-Diploid Colorectal Cancer: A Link between Numbers and Structure
Source: PLoS One. 2008 Feb 20;3(2):e1632. doi: 10.1371/journal.pone.0001632 (PMC2238794; doi:10.1371/journal.pone.0001632)
Supplement: Table S4 — Analysis of the two-by-two associations between 29 chromosomal imbalances in the sample of 96 near-diploid colorectal tumors Observed (A) and theoretical (B) numbers of tumors that display (above the diagonal) or not (below the diagonal) each two-by-two association. Theoretical values were calculated under the hypothesis that associations occurred only by chance according to the relative frequency of each alteration. In C are indicated in each box the Chi2 values (cut-off value = 3.84, p<0.05) testing the observed number of tumors that display each association compared to the theoretical value. Forty-five associations were observed more frequently than expected by chance (positive associations), among them associations between chromosomal losses (blue background), chromosomal gains (pink background), or chromosomal gains and losses (purple background) are distinguished, and 6 were too rarely observed (negative associations, yellow background). NO: no object, *theoretical value is inferior to 1. The first row in italics in B indicates the frequency of each aberration. (0.12 MB PDF) [file pone.0001632.s004.pdf]

A

	-1p	-5q	-6q	+7	-8p	+8q	-10q	+12	+13	+13q	-14	-15	-17p	+17q	-18	+20	+X	-Y	-4	-6	+6	-8	+8	-4p	-4q	-11p	-11q	-9p	-9q
**-1p**	NQ	7	4	6	12	8	5	3	9	9	11	7	17	6	24	10	5	4	11	1	0	3	4	3	2	3	5	4	2
**-5q**	89	NQ	3	2	7	4	3	1	3	4	3	2	8	2	9	6	2	1	3	0	1	1	0	1	3	2	2	1	1
**-6q**	92	93	NQ	2	5	3	1	2	3	2	3	2	7	1	8	4	4	1	3	0	0	2	3	0	0	1	1	1	1
**+7**	90	94	94	NQ	10	13	2	15	18	4	3	2	13	6	13	20	15	7	2	0	6	2	4	2	2	2	1	2	1
**-8p**	84	89	91	86	NQ	20	6	2	8	7	11	10	20	4	25	9	9	6	8	0	4	0	0	4	2	2	2	5	2
**+8q**	88	92	93	83	76	NQ	4	5	11	5	8	9	18	5	18	11	11	6	4	0	6	0	0	3	1	1	2	3	2
**-10q**	91	93	95	94	90	92	NQ	2	3	3	2	6	5	1	9	4	1	5	2	0	0	0	0	1	2	0	0	2	0
**+12**	93	95	94	81	94	91	94	NQ	13	1	1	0	3	2	6	14	9	3	1	1	6	1	5	0	0	1	1	0	0
**+13**	87	93	93	78	88	85	93	83	NQ	1	5	3	12	5	15	20	15	4	4	1	6	1	5	1	0	0	2	1	2
**+13q**	87	92	94	92	89	91	93	95	95	NQ	9	7	11	0	15	5	3	2	3	1	0	3	0	0	2	3	2	3	0
**-14**	85	93	93	93	85	88	94	95	91	87	NQ	8	16	3	19	3	2	3	5	1	1	4	0	0	1	1	4	4	2
**-15**	89	94	94	94	86	87	90	96	93	89	88	NQ	10	1	17	3	2	3	2	0	0	2	0	3	3	1	1	1	1
**-17p**	79	88	89	83	76	78	91	93	84	85	80	86	NQ	10	33	16	16	7	9	2	5	3	3	4	5	2	5	4	4
**+17q**	90	94	95	90	92	91	95	94	91	96	93	95	86	NQ	7	5	5	3	1	0	2	1	2	1	1	0	1	0	1
**-18**	72	87	88	83	71	78	87	90	81	81	77	79	63	89	NQ	21	17	9	13	2	5	6	3	5	5	4	5	5	3
**+20**	86	90	92	76	87	85	92	82	76	91	93	93	80	91	75	NQ	17	5	4	0	4	2	4	2	2	2	2	1	2
**+X**	91	94	92	81	87	85	95	87	81	93	94	94	80	91	79	79	NQ	6	2	1	6	1	4	1	1	1	0	1	2
**-Y**	31	34	34	28	29	29	30	32	31	33	32	32	28	32	26	30	29	NQ	3	1	2	1	1	1	1	0	1	1	1
**-4**	85	93	93	94	88	92	94	95	92	93	91	94	87	95	83	92	94	93	NQ	1	2	1	1	0	0	2	5	2	2
**-6**	95	96	96	96	96	96	96	95	95	95	95	96	94	96	94	96	95	95	95	NQ	NO	0	0	0	0	0	1	0	2
**+6**	96	95	96	90	92	90	96	90	90	96	95	96	91	94	91	92	90	94	94	NO	NQ	0	2	0	1	0	0	1	0
**-8**	93	95	94	94	96	96	96	95	95	93	92	94	93	95	90	94	95	95	95	96	96	NQ	NO	0	0	1	1	0	1
**+8**	92	96	93	92	96	96	96	91	91	96	96	96	93	94	93	92	92	95	95	96	94	NO	NQ	0	0	1	0	0	0
**-4p**	93	95	96	94	92	93	95	96	95	96	96	93	92	95	91	94	95	95	96	96	96	96	96	NQ	0	0	0	0	0
**-4q**	94	93	96	94	94	95	94	96	96	94	95	93	91	95	91	94	95	95	96	96	96	96	96	96	NQ	0	1	1	1
**-11p**	93	94	95	94	94	95	96	95	96	93	95	95	94	96	92	94	95	96	94	96	96	95	95	96	96	NQ	0	0	0
**-11q**	91	94	95	95	94	94	96	95	94	94	92	95	91	95	91	94	96	95	91	95	96	96	96	96	95	96	NQ	0	2
**-9p**	92	95	95	94	91	93	94	96	95	93	92	95	92	96	91	95	95	95	94	96	95	96	96	96	95	96	96	NQ	0
**-9q**	94	95	95	95	94	94	96	96	94	96	94	95	92	95	93	94	94	95	94	96	96	95	96	96	95	96	94	96	NQ

B

	-1p	-5q	-6q	+7	-8p	+8q	-10q	+12	+13	+13q	-14	-15	-17p	+17q	-18	+20	+X	-Y	-4	-6	+6	-8	+8	-4p	-4q	-11p	-11q	-9p	-9q
	29.16	*12.50*	*11.45*	*33.33*	*29.16*	*27.08*	*11.46*	*22.92*	*33.33*	*18.75*	*19.79*	*17.71*	*41.66*	*12.50*	*53.12*	*37.50*	*28.12*	*37.14*	*13.54*	*3.13*	*10.42*	*6.25*	*11.46*	*5.21*	*6.25*	*5.21*	*6.25*	*6.25*	*4.17*
**-1p**	NQ	3.50	3.21	9.33	8.16	7.58	3.21	6.42	9.33	5.25	5.54	4.96	11.66	3.50	14.87	10.50	7.87	3.79	3.79	0.87	2.92	1.75	3.21	1.46	1.75	1.46	1.75	1.75	1.17
**-5q**	92.50	NQ	1.37	4.00	3.50	3.25	1.38	2.75	4.00	2.25	2.37	2.13	5.00	1.50	6.37	4.50	3.37	1.62	1.63	0.38	1.25	0.75	1.38	0.63	0.75	0.63	0.75	0.75	0.5
**-6q**	92.79	94.63	NQ	3.66	3.21	2.98	1.26	2.52	3.66	2.06	2.18	1.95	4.58	1.37	5.84	4.12	3.09	1.49	1.49	0.34	1.15	0.69	1.26	0.57	0.69	0.57	0.69	0.69	0.46
**+7**	86.67	92.00	92.34	NQ	9.33	8.66	3.67	7.33	10.66	6.00	6.33	5.67	13.33	4.00	17.00	12.00	9.00	4.33	4.33	1.00	3.33	2.00	3.67	1.67	2.00	1.67	2.00	2.00	1.33
**-8p**	87.84	92.50	92.79	86.67	NQ	7.58	3.21	6.42	9.33	5.25	5.54	4.96	11.66	3.50	14.87	10.50	7.87	3.79	3.79	0.87	2.92	1.75	3.21	1.46	1.75	1.46	1.75	1.75	1.17
**+8q**	88.42	92.75	93.02	87.34	88.42	NQ	2.98	5.96	8.66	4.87	5.14	4.60	10.83	3.25	13.81	9.75	7.31	3.52	3.52	0.81	2.71	1.62	2.98	1.35	1.62	1.35	1.62	1.62	1.08
**-10q**	92.79	94.62	94.74	92.33	92.79	93.02	NQ	2.52	3.67	2.06	2.18	1.95	4.58	1.38	5.84	4.13	3.09	1.49	1.49	0.34	1.15	0.69	1.26	0.57	0.69	0.57	0.69	0.69	0.46
**+12**	89.58	93.25	93.48	88.67	89.58	90.04	93.48	NQ	7.33	4.13	4.35	3.90	9.17	2.75	11.69	8.25	6.19	2.98	2.98	0.69	2.29	1.38	2.52	1.15	1.38	1.15	1.38	1.38	0.92
**+13**	86.67	92.00	92.34	85.34	86.67	87.34	92.33	88.67	NQ	6.00	6.33	5.67	13.33	4.00	17.00	12.00	9.00	4.33	4.33	1.00	3.33	2.00	3.67	1.67	2.00	1.67	2.00	2.00	1.33
**+13q**	90.75	93.75	93.94	90.00	90.75	91.13	93.94	91.87	90.00	NQ	3.56	3.19	7.50	2.25	9.56	6.75	5.06	2.44	2.44	0.56	1.88	1.13	2.06	0.94	1.13	0.94	1.13	1.13	0.75
**-14**	90.46	93.63	93.82	89.67	90.46	90.86	93.82	91.65	89.67	92.44	NQ	3.36	7.91	2.37	10.09	7.12	5.34	2.57	2.57	0.59	1.98	1.19	2.18	0.99	1.19	0.99	1.19	1.19	0.79
**-15**	91.04	93.87	94.05	90.33	91.04	91.40	94.05	92.10	90.33	92.81	92.64	NQ	7.08	2.13	9.03	6.38	4.78	2.30	2.30	0.53	1.77	1.06	1.95	0.89	1.06	0.89	1.06	1.06	0.71
**-17p**	84.34	91.00	91.42	82.67	84.34	85.17	91.42	86.83	82.67	88.50	88.09	88.92	NQ	5.00	21.24	15.00	11.25	5.42	5.42	1.25	4.17	2.50	4.58	2.08	2.50	2.08	2.50	2.50	1.67
**+17q**	92.50	94.50	94.63	92.00	92.50	92.75	94.62	93.25	92.00	93.75	93.63	93.87	91.00	NQ	6.37	4.50	3.37	1.62	1.63	0.38	1.25	0.75	1.38	0.63	0.75	0.63	0.75	0.75	0.5
**-18**	81.13	89.63	90.16	79.00	81.13	82.19	90.16	84.31	79.00	86.44	85.91	86.97	74.76	89.63	NQ	19.12	14.34	6.91	6.91	1.59	5.31	3.19	5.84	2.66	3.19	2.66	3.19	3.19	2.12
**+20**	85.50	91.50	91.88	84.00	85.50	86.25	91.87	87.75	84.00	89.25	88.88	89.62	81.00	91.50	76.88	NQ	10.12	4.87	4.88	1.13	3.75	2.25	4.13	1.88	2.25	1.88	2.25	2.25	1.5
**+X**	88.13	92.63	92.91	87.00	88.13	88.69	92.91	89.81	87.00	90.94	90.66	91.22	84.75	92.63	81.66	85.88	NQ	3.66	3.66	0.84	2.81	1.69	3.09	1.41	1.69	1.41	1.69	1.69	1.12
**-Y**	31.21	33.38	33.51	30.67	31.21	31.48	33.51	32.02	30.67	32.56	32.43	32.70	29.58	33.38	28.09	30.13	31.34	NQ	4.83	1.11	3.71	2.23	4.09	1.86	2.23	1.86	2.23	2.23	1.48
**-4**	92.21	94.38	94.51	91.67	92.21	92.48	94.51	93.02	91.67	93.56	93.43	93.70	90.58	94.38	89.09	91.13	92.34	91.17	NQ	0.41	1.35	0.81	1.49	0.68	0.81	0.68	0.81	0.81	0.54
**-6**	95.13	95.63	95.66	95.00	95.13	95.19	95.66	95.31	95.00	95.44	95.41	95.47	94.75	95.63	94.41	94.88	95.16	94.89	95.59	NQ	NO	0.19	0.34	0.16	0.19	0.16	0.19	0.19	0.12
**+6**	93.08	94.75	94.86	92.67	93.08	93.29	94.85	93.71	92.67	94.13	94.02	94.23	91.83	94.75	90.69	92.25	93.19	92.29	94.65	NO	NQ	0.63	1.15	0.52	0.63	0.52	0.63	0.63	0.42
**-8**	94.25	95.25	95.31	94.00	94.25	94.38	95.31	94.62	94.00	94.88	94.81	94.94	93.50	95.25	92.81	93.75	94.31	93.77	95.19	95.81	95.38	NQ	NO	0.31	0.38	0.31	0.38	0.38	0.25
**+8**	92.79	94.63	94.74	92.33	92.79	93.02	94.74	93.48	92.33	93.94	93.82	94.05	91.42	94.63	90.16	91.88	92.91	91.91	94.51	95.66	94.85	NO	NQ	0.57	0.69	0.57	0.69	0.69	0.46
**-4p**	94.54	95.38	95.43	94.33	94.54	94.65	95.43	94.85	94.33	95.06	95.01	95.11	93.92	95.38	93.34	94.13	94.59	94.14	95.32	95.84	95.48	95.69	95.43	NQ	0.31	0.26	0.31	0.31	0.21
**-4q**	94.25	95.25	95.31	94.00	94.25	94.38	95.31	94.62	94.00	94.88	94.81	94.94	93.50	95.25	92.81	93.75	94.31	93.77	95.19	95.81	95.38	95.63	95.31	95.69	NQ	0.31	0.38	0.38	0.25
**-11p**	94.54	95.38	95.43	94.33	94.54	94.65	95.43	94.85	94.33	95.06	95.01	95.11	93.92	95.38	93.34	94.13	94.59	94.14	95.32	95.84	95.48	95.69	95.43	95.74	95.69	NQ	0.31	0.31	0.21
**-11q**	94.25	95.25	95.31	94.00	94.25	94.38	95.31	94.62	94.00	94.88	94.81	94.94	93.50	95.25	92.81	93.75	94.31	93.77	95.19	95.81	95.38	95.63	95.31	95.69	95.63	95.69	NQ	0.38	0.25
**-9p**	94.25	95.25	95.31	94.00	94.25	94.38	95.31	94.62	94.00	94.88	94.81	94.94	93.50	95.25	92.81	93.75	94.31	93.77	95.19	95.81	95.38	95.63	95.31	95.69	95.63	95.69	95.63	NQ	0.25
**-9q**	94.83	95.50	95.54	94.67	94.83	94.92	95.54	95.08	94.67	95.25	95.21	95.29	94.33	95.50	93.88	94.50	94.88	94.51	95.46	95.88	95.58	95.75	95.54	95.79	95.75	95.79	95.75	95.75	NQ

C

	-1p	-5q	-6q	+7	-8p	+8q	-10q	+12	+13	+13q	-14	-15	-17p	+17q	-18	+20	+X	-Y	-4	-6	+6	-8	+8	-4p	-4q	-11p	-11q	-9p	-9q
**-1p**	NO	3.63	0.20	1.32	1.97	0.03	1.04	1.95	0.01	2.84	5.71	0.89	2.78	1.85	6.63	0.03	1.14	0.01	12.52	0.02	4.06	0.91	0.20	1.66	0.04	1.66	4.47	2.95	0.60
**-5q**		NO	1.95	1.04	3.63	0.18	1.95	1.15	0.26	1.39	0.17	0.01	1.90	0.17	1.16	0.52	0.58	0.25	1.18	0.38	0.05	0.08	1.39	0.23	6.80*	3.04	2.10	0.08	0.50
**-6q**			NO	0.79	1.04	0.00	0.05	0.11	0.12	0.00	0.32	0.00	1.34	0.10	0.85	0.00	0.28	0.17	1.56	0.34	1.16	2.53	2.44	0.58	0.69	0.32	0.14	0.14	0.64
**+7**				NO	0.05	2.38	0.79	8.68	5.68	0.71	1.88	2.52	0.01	1.04	1.14	6.10	4.42	1.87	1.32	1.01	2.21	0.00	0.03	0.00	0.07	0.51	0.00	0.08	
**-8p**					NO	22.09	2.51	3.26	0.21	0.62	5.71	4.50	6.79	0.07	8.17	0.24	0.18	1.44	4.87	0.88	0.42	1.78	4.36	4.50	0.04	0.20	0.04	4.47	0.60
**+8q**						NO	0.36	0.16	0.69	0.00	1.67	4.41	5.35	0.98	1.49	0.18	2.02	1.94	0.07	0.82	4.12	1.65	4.13	2.03	0.24	0.09	0.09	1.18	0.78
**-10q**							NO	0.11	0.13	0.44	0.01	6.69	0.04	0.10	1.81	0.00	1.46	6.56	0.18	0.35	2.21	0.69	1.28	0.32	2.52	0.58	0.69	2.52	0.46
**+12**								NO	4.74	2.47	2.71	5.09	4.59	0.21	3.15	4.38	1.37	0.00	1.36	0.14	6.15	0.10	2.50	1.16	1.40	0.02	0.10	1.40	0.93
**+13**									NO	4.44	0.30	1.33	0.15	0.26	0.29	6.10	4.42	0.03	0.03	0.00	2.21	0.51	0.50	0.27	2.04	1.70	0.00	0.51	0.34
**+13q**										NO	8.62	4.72	1.77	2.30	3.44	0.49	0.89	0.08	0.13	0.34	1.91	3.16	2.11	0.95	0.69	4.58*	0.69	3.16	0.76
**-14**											NO	5.37	9.00	0.17	8.79	2.58	2.21	0.08	2.35	0.28	0.49	4.62	2.23	1.00	0.03	0.00	4.62	4.62	1.86
**-15**												NO	1.30	0.61	7.76	1.91	1.70	0.23	0.04	0.53	1.80	0.84	1.99	5.10*	3.57	0.01	0.00	0.00	0.12
**-17p**													NO	4.38	8.35	0.08	2.28	0.55	2.51	0.46	0.17	0.10	0.57	1.80	2.57	0.00	2.57	0.92	3.33
**+17q**														NO	0.07	0.06	0.81	1.22	0.24	0.38	0.46	0.08	0.29	0.23	0.08	0.63	0.08	0.76	0.50
**-18**															NO	0.23	0.58	0.79	5.80	0.11	0.02	2.57	1.47	2.13	1.07	0.70	1.07	1.07	0.37
**+20**																NO	5.22	0.00	0.17	1.14	0.02	0.03	0.00	0. 01	0.03	0.01	0.03	0.71	0.17
**+X**																	NO	1.68	0.78	0.03	3.72	0.28	0.27	0.12	0.28	0.12	1.72	0.28	0.69
**-Y**																		NO	0.73	0.01	0.82	0.69	2.43	0.40	0.69	1.89	0.69	0.69	0.16
**-4**																			NO	0.87	0.31	0.04	0.16	0.68	0.82	2.60	21.8*	1.75	3.95
**-6**																				NO	NO	0.19	0.34	0.16	0.19	0.16	3.53	0.19	28.2*
**+6**																					NO	0.63	0.64	0.52	0.23	0.52	0.63	0.23	0.42
**-8**																						NO	NO	0.31	0.38	1.52	1.05	0.38	2.26
**+8**																							NO	0.58	0.69	0.32	0.69	0.69	0.46
**-4p**																								NO	0.31	0.26	0.31	0.31	0.21
**-4q**																									NO	0.31	1.05	1.05	2.26
**-11p**																										NO	0.31	0.31	0.21
**-11q**																											NO	0.38	12.3*
**-9p**																												NO	0.25
**-9q**																													NO

Legend:
- Positive association between chromosomal losses (blue)
- Positive association between chromosomal gains (pink)
- Positive association between chromosomal gains and losses (purple)
- Negative association (yellow)

Supplementary Table S4. Analysis of the two-by-two associations between 29 chromosomal imbalances in the sample of 96 near-diploid colorectal tumors

Observed (A) and theoretical (B) numbers of tumors that display (above the diagonal) or not (below the diagonal) each two-by-two association. Theoretical values were calculated under the hypothesis that associations occurred only by chance according to the relative frequency of each alteration. In C are indicated in each box the Chi2 values (cut-off value=3.84, p<0.05) testing the observed number of tumors that display each association compared to the theoretical value. Forty-five associations were observed more frequently than expected by chance (positive associations), among them associations between chromosomal losses (blue background), chromosomal gains (pink background), or chromosomal gains and losses (purple background) are distinguished, and 6 were too rarely observed (negative associations, yellow background). NO: no object, *theoretical value is inferior to 1. The first row in italics in B indicates the frequency of each aberration.
