# Supplementary material for: Chromosomal Instability in Near-Diploid Colorectal Cancer: A Link between Numbers and Structure
Source: PLoS One. 2008 Feb 20;3(2):e1632. doi: 10.1371/journal.pone.0001632 (PMC2238794; doi:10.1371/journal.pone.0001632)
Supplement: Figure S1 — R-banding schematic karyotype indicating the chromosomal imbalances resulting from the structural rearrangements identified in our sample of 96 near-diploid colorectal cancers. Red lines, gains; blue lines, losses; thick red lines, multiple gains; dotted blue lines, deletions within a chromosomal region that could not be accurately identified; numbers, case number. (0.09 MB PDF) [file pone.0001632.s005.pdf]

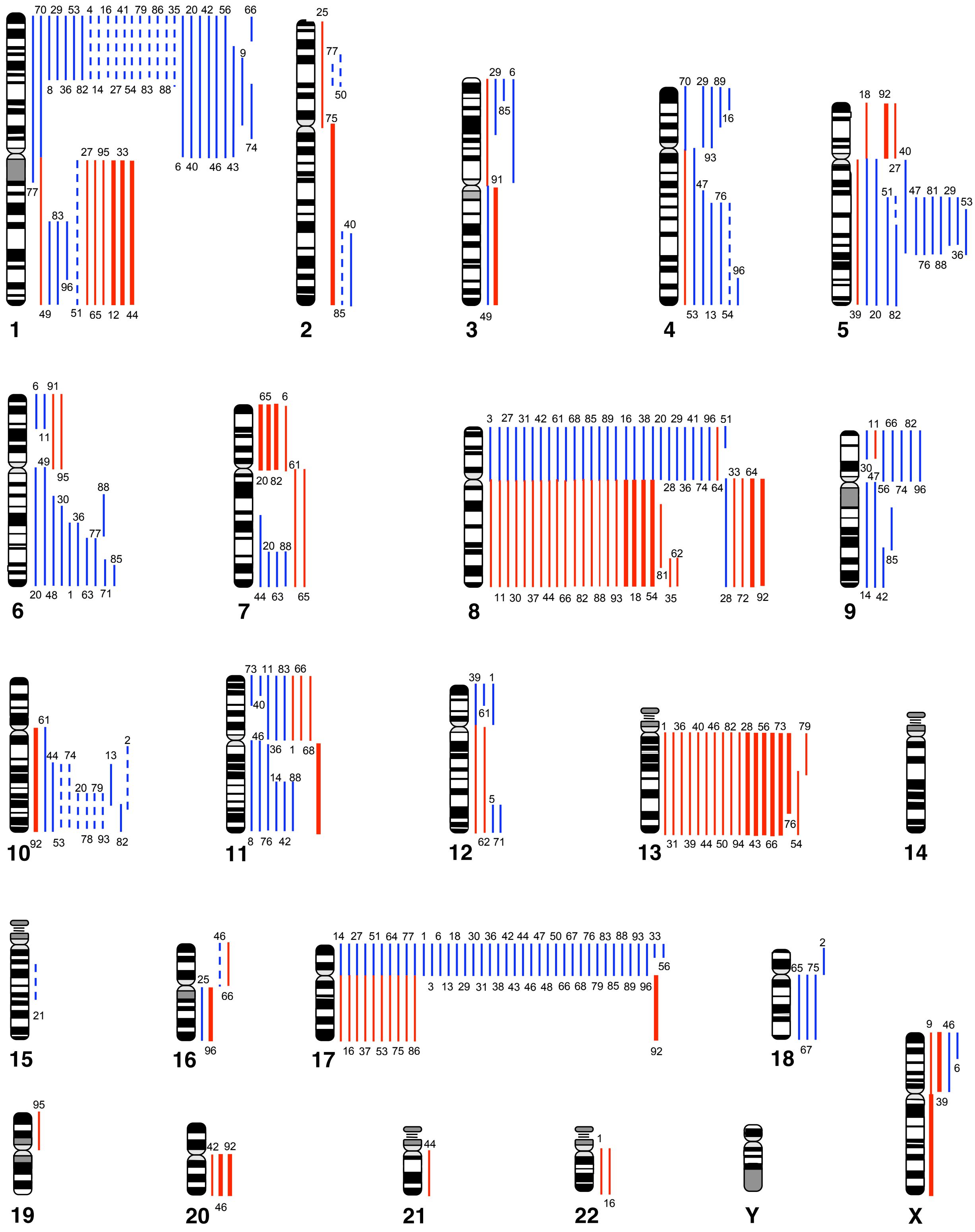


**Supplementary Figure S1** R-banding schematic karyotype indicating the chromosomal imbalances resulting from the structural rearrangements identified in our sample of 96 near-diploid colorectal cancers.
Red lines, gains; blue lines, losses; thick red lines, multiple gains; dotted blue lines, deletions within a chromosomal region that could not be accurately identified; numbers, case number.
